# Nod Factor Effects on Root Hair-Specific Transcriptome of *Medicago truncatula*: Focus on Plasma Membrane Transport Systems and Reactive Oxygen Species Networks

**DOI:** 10.3389/fpls.2016.00794

**Published:** 2016-06-07

**Authors:** Isabelle Damiani, Alice Drain, Marjorie Guichard, Sandrine Balzergue, Alexandre Boscari, Jean-Christophe Boyer, Véronique Brunaud, Sylvain Cottaz, Corinne Rancurel, Martine Da Rocha, Cécile Fizames, Sébastien Fort, Isabelle Gaillard, Vincent Maillol, Etienne G. J. Danchin, Hatem Rouached, Eric Samain, Yan-Hua Su, Julien Thouin, Bruno Touraine, Alain Puppo, Jean-Marie Frachisse, Nicolas Pauly, Hervé Sentenac

**Affiliations:** ^1^Centre National de la Recherche Scientifique, Institut National de la Recherche Agronomique, UMR 1355-7254 Institut Sophia Agrobiotech, Université Nice Sophia AntipolisSophia Antipolis, France; ^2^Biochimie and Physiologie Moléculaire des Plantes, UMR 5004 Centre National de la Recherche Scientifique/386 Institut National de la Recherche Agronomique/SupAgro Montpellier/Université de Montpellier, Campus SupAgro-Institut National de la Recherche AgronomiqueMontpellier, France; ^3^Institute for Integrative Biology of the Cell, CEA, Centre National de la Recherche Scientifique, Université Paris-Sud, Université Paris-SaclayGif sur Yvette, France; ^4^POPS Transcriptomic Platform, Centre National de la Recherche Scientifique, Institute of Plant Sciences Paris-Saclay, Institut National de la Recherche Agronomique, Université Paris-Sud, Université Evry, Université Paris-SaclayOrsay, France; ^5^POPS Transcriptomic Platform, Institute of Plant Sciences Paris-Saclay, Paris DiderotOrsay, France; ^6^Université Grenoble Alpes, CERMAVGrenoble, France; ^7^Centre National de la Recherche Scientifique, CERMAVGrenoble, France; ^8^Laboratoire d'Informatique, de Robotique et de Microélectronique de Montpellier and Institut de Biologie Computationnelle, Centre National de la Recherche Scientifique and Université MontpellierMontpellier, France; ^9^State Key Laboratory of Soil and Sustainable Agriculture, Institute of Soil Science, Chinese Academy of SciencesNanjing, China

**Keywords:** *Medicago truncatula*, root hairs, deep-RNA sequencing, Nod factors (lipochitooligosaccharides), legume-rhizobium symbiosis, plasma membrane transport systems, reactive oxygen species

## Abstract

Root hairs are involved in water and nutrient uptake, and thereby in plant autotrophy. In legumes, they also play a crucial role in establishment of rhizobial symbiosis. To obtain a holistic view of *Medicago truncatula* genes expressed in root hairs and of their regulation during the first hours of the engagement in rhizobial symbiotic interaction, a high throughput RNA sequencing on isolated root hairs from roots challenged or not with lipochitooligosaccharides Nod factors (NF) for 4 or 20 h was carried out. This provided a repertoire of genes displaying expression in root hairs, responding or not to NF, and specific or not to legumes. In analyzing the transcriptome dataset, special attention was paid to pumps, transporters, or channels active at the plasma membrane, to other proteins likely to play a role in nutrient ion uptake, NF electrical and calcium signaling, control of the redox status or the dynamic reprogramming of root hair transcriptome induced by NF treatment, and to the identification of papilionoid legume-specific genes expressed in root hairs. About 10% of the root hair expressed genes were significantly up- or down-regulated by NF treatment, suggesting their involvement in remodeling plant functions to allow establishment of the symbiotic relationship. For instance, NF-induced changes in expression of genes encoding plasma membrane transport systems or disease response proteins indicate that root hairs reduce their involvement in nutrient ion absorption and adapt their immune system in order to engage in the symbiotic interaction. It also appears that the redox status of root hair cells is tuned in response to NF perception. In addition, 1176 genes that could be considered as “papilionoid legume-specific” were identified in the *M. truncatula* root hair transcriptome, from which 141 were found to possess an ortholog in every of the six legume genomes that we considered, suggesting their involvement in essential functions specific to legumes. This transcriptome provides a valuable resource to investigate root hair biology in legumes and the roles that these cells play in rhizobial symbiosis establishment. These results could also contribute to the long-term objective of transferring this symbiotic capacity to non-legume plants.

## Introduction

Root hairs are long tubular outgrowths that project into the soil from root epidermal cells named trichoblasts. Together with the pollen tube in plants, axons in animals, and hyphae in filamentous fungi, they provide one of the very rare models of cell types displaying polarized “tip” growth. Root hair elongation results in an increased area of the root-soil interface. At this interface, root hairs contribute to plant autotrophy by taking up nutrient ions and water from the soil solution.

Root hairs are also involved in beneficial interactions with soil microorganisms. They exudate compounds that act as a chemotactic signal or promote the growth of symbiotic fungi and bacteria (Bais et al., 2006). Moreover, they are directly involved in the formation of nitrogen-fixing nodules in legumes. The plant secretes signaling flavonoid compounds that are perceived by the rhizobial symbiont, which responds to this message by secreting specific lipochitooligosaccharides, named Nod factors (NF; Oldroyd and Downie, 2008). NF are signal molecules whose binding to root hair receptors triggers complex signaling events leading the root hair to curl and thereby to entrap rhizobia. Then, an infection thread develops, allowing rhizobia to migrate through the root cortex toward the nodule primordium.

With respect to the initial signals triggered by NF perception, the earliest events that have been reported so far involve reactive oxygen species (ROS) and ion fluxes across the cell membrane. ROS signals have been recorded within seconds after addition of NF on growing root hairs (Cardenas et al., 2008). In contrast, several minutes after NF perception, the production of hydrogen peroxide (H_2_O_2_) appears to be inhibited (Shaw and Long, 2003; Lohar et al., 2007). Several hours later, a gradual increase in ROS production occurs in root cortical cells of inoculated plants, which peaks at 24 h after rhizobial inoculation and remains high 48 h after inoculation (Ramu et al., 2002; Peleg-Grossman et al., 2007, 2012). Thus, ROS play a role in the early signaling leading to establishment of the symbiotic partnership (Montiel et al., 2012) as well as later on, when rhizobia invade the root hair via progression of the infection thread (for review, see Puppo et al., 2013).

Ions whose transport across the root hair plasma membrane has been shown to be rapidly modulated during the initial root hair response to NF are H^+^, Ca^2+^, Cl^−^, and K^+^ (Felle et al., 1996, 1998, 1999; Kurkdjian et al., 2000). In summary, the present model is that NF perception leads to a transient inhibition of plasma membrane H^+^-ATPases (proton pumps), an activation of Ca^2+^ channels, allowing a depolarizing influx of Ca^2+^, immediately followed by an activation of anion (Cl^−^ permeable) channels, mediating an efflux of anions that further depolarizes the cell membrane. In turn, this depolarization activates voltage-gated K^+^ channels, giving rise to a repolarizing efflux of K^+^. This series of events is thought to generate electrical and calcium signals that play a major role in the initial dialogue between root hairs and rhizobia (Felle et al., 1996, 1998, 1999; Kurkdjian et al., 2000). It might be connected to the first ROS signals triggered by NF perception since ROS production by NADPH oxidases has been shown to stimulate plasma membrane Ca^2+^ channel activity in Arabidopsis root hairs (Foreman et al., 2003). Also, based on results obtained in Arabidopsis, ROS could play a role in control of K^+^ channel activity involved in electrical signaling (Garcia-Mata et al., 2010; Tran et al., 2013) as well as in control of annexins involved in Ca^2+^ signaling (Richards et al., 2014). The decision of the plant to engage in rhizobial symbiosis is strongly dependent on the availability of nutrient ions in the soil solution, in particular of nitrate and ammonium (Barbulova et al., 2007). Hence, by routing nutrient ions, transport systems are likely to impact the plant decision to establish symbiosis. ROS signals are likely to play a role in this process since nutrient ion deprivation has been shown to lead to altered levels of ROS in Arabidopsis and *M. truncatula* roots (Schachtman and Shin, 2007; Bonneau et al., 2013), and NADPH oxidases genes are necessary for up-regulation of genes in response to nutrient deficiency (Shin and Schachtman, 2004).

The multiple and essential functions of the root hair clearly establish this cell type as a system biology model to investigate plant cell development, uptake of water and nutrients, and response to abiotic and biotic signals. Indeed, a number of genome-wide studies have been published using root hairs from several model and crop plants (Hossain et al., 2015; Wang et al., 2016). The present paper is conceived as a contribution to improved understanding of the molecular mechanisms underpinning the roles of root hairs in legumes. We used *M. truncatula* as a model legume. A detailed analysis of gene expression in *M. truncatula* root hairs from young seedlings (without lateral roots) and of their responses to NF treatments for 4 h (to get information on early NF signaling events) or 20 h has been carried out using a high throughput RNA sequencing strategy (RNA-seq). This is likely to provide the first RNA-seq dataset from *M. truncatula* root hairs, thereby the most exhaustive transcriptome available to date for this cell type, and thus a valuable resource to investigate root hair functions in legumes. Expert gene analyses have been focused in order to uncover ROS networks and pump, transporter and channel machineries likely to play a role in two major functions of root hairs, namely nutrient ion uptake and early molecular dialogue with rhizobia following NF perception. Moreover, using whole genome comparisons, we identified legume-specific genes expressed in root hairs, responding or not to NF. Altogether, these results constitute a promising dataset for researchers interested in plant mineral nutrition, rhizobial symbiosis, and legume specificities.

## Results

### Preparation of root hair libraries

**Figure 1:**
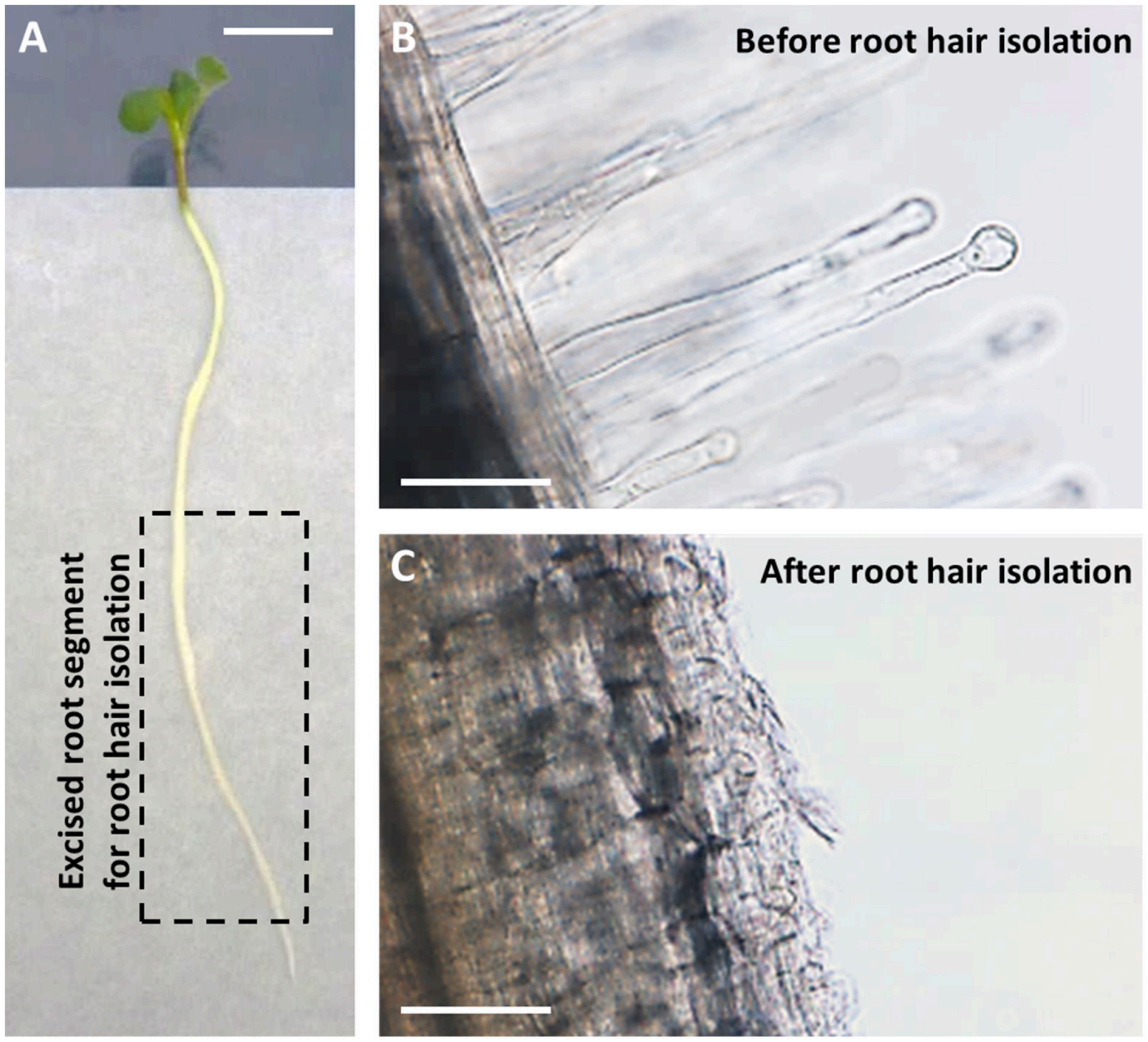
**Root hairs isolation from *Medicago truncatula*. (A)** Five-day old seedling just before root hairs isolation (Scale bar = 1 cm). Root hairs were isolated by vortexing frozen excised root segments long of about 4 cm and free from the root tip (0.5 cm) as displayed by the dotted line rectangle. **(B,C)** Representative photographs of root material before **(B)** and after **(C)** root vortexing, showing the absence of root hairs in the remaining material at the end of the isolation procedure (Scale bar = 100 μm).

**Figure 2:**
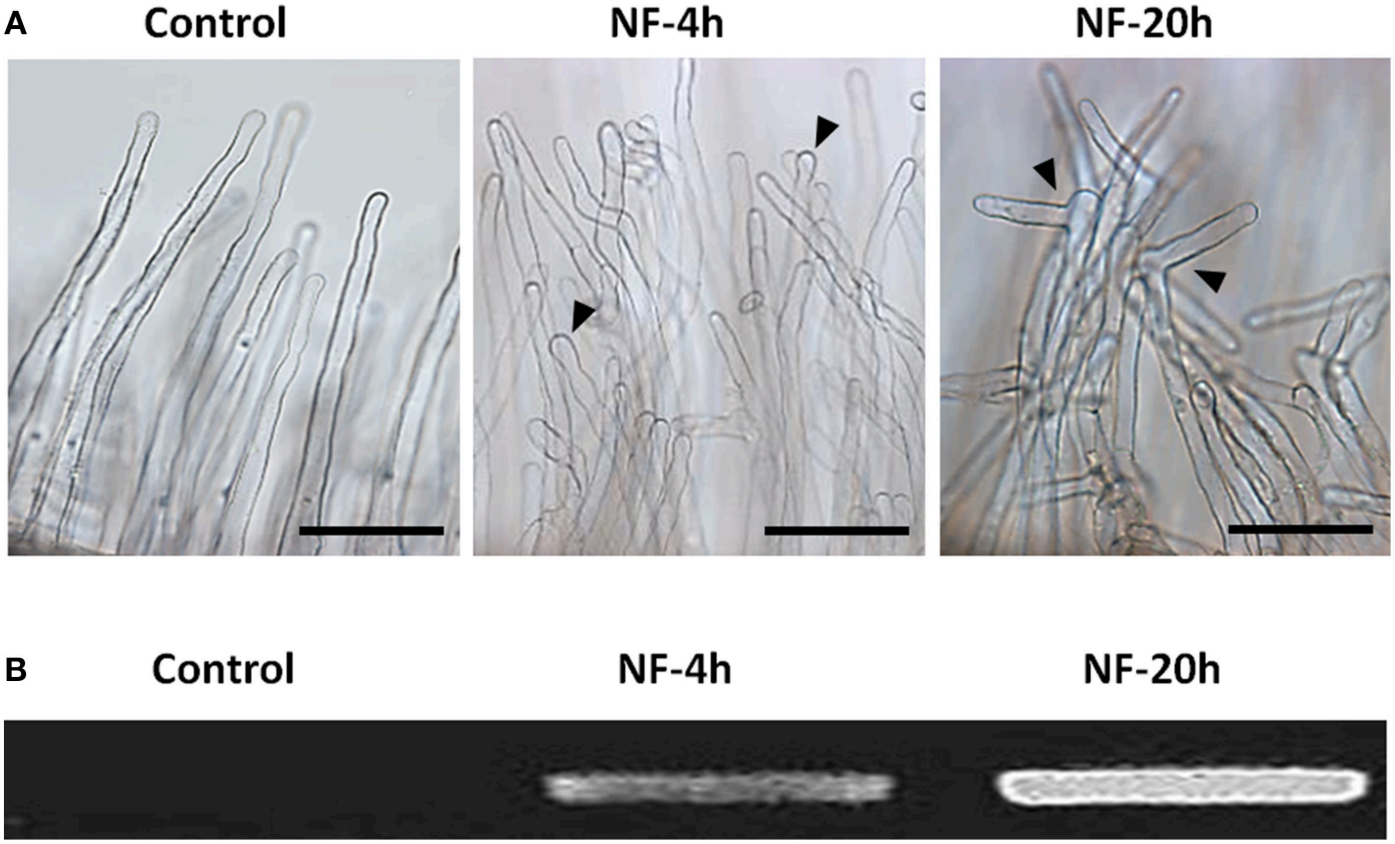
**Root responses to Nod factor treatments. (A)** Root hair morphological responses. Representative photographs of root hairs from roots subjected to a control treatment with pure H_2_O or treated with 10 nM Nod factors (NF) for 4 h or 20 h (left, middle, and right panel, respectively; Scale bar = 100 μm). **(B)** Gene expression response to NF highlighted by increased *MtENOD11* transcript accumulation. Differences in *MtENOD11* transcript levels were revealed by RT-PCR experiments performed in parallel on control roots or roots pretreated by Nod factors for 4 or 20 h (left, middle, and right lane of the electrophoresis gel, respectively).

Root hairs were obtained from excised root segments (Figure 1). Before excision, the root systems were treated with 10 nM NF solution for 4 or 20 h, or with pure H_2_O as a control treatment (NF 4 h, NF 20 h, and control treatments, respectively). Microscopic observation of root segment samples before root hair isolation indicated that the two NF treatments had actually induced the expected root hair morphological changes, i.e., root hair tip swelling in the case of the NF 4 h treatment, and reoriented root hair growth and branching in the case of the NF 20 h treatment (Figure 2A). Root hairs in the H_2_O treated control plants did not display such features (Figure 2A). RT-PCR analyses performed on root segments free from root hairs (material remaining after root hair isolation) provided further evidence that the NF treatments had been effective by showing a strong accumulation of *MtENOD11* transcripts (Figure 2B), one of the earliest markers induced by NF perception in root epidermal cells (Journet et al., 2001). Finally, absence of transcripts from the *MtSUNN* gene, which is known to be specifically expressed in root stele tissues (Schnabel et al., 2005), indicated that the root hair RNA pools were not substantially contaminated by RNA from inner root cells, and thus from whole root segments.

### General features of the *M. truncatula* root hair transcriptome

RNA sequencing was carried out on two independent biological replicates for each of the three treatments (H_2_O, NF 4 h, NF 20 h), yielding six root hair transcriptomic datasets. We generated ~366 million high-quality paired-end reads, which were mapped to the *M. truncatula* predicted transcriptome (Mt4.0v1). About 266 million (ca. 73%) of paired-end reads were unambiguously mapped (Table S1). In order to assess the experimental variability, the two replicates obtained for a given treatment were compared by plotting the expression levels (expressed in Count per Million Reads) found in one dataset against those found in the second dataset (Figure S1). Linear regression analysis yielded *R*^2^ coefficient of 0.99, 0.99, and 0.97 for the control, NF 4 h, and NF 20 h treatments, respectively. For a large majority of genes (≥65% whatever the treatment), the fold change in expression level between the two repetitions was lower than 1.5. As could be anticipated, we noticed that the biological variability in terms of expression level fold change was larger for genes displaying low expression levels. In order to further assess consistency among biological replicates for the transcriptome datasets, we subjected the expression data derived from the six datasets to principal component analysis (PCA; Figure S2). The first component (Dim1) clearly separated the control from the NF treated samples (Figure S2A) and the overall distribution confirmed that there was no biological repetition bias (Figure S2B).

To validate RNA-seq based differential gene expression in response to NF treatments, quantitative real-time RT-PCR (RT-qPCR) tests were also carried out. A set of 20 genes was selected (Table S11), amongst which receptors, pumps, and ion channels differing in their levels of expression. The expression level provided by the RT-qPCR experiments for the NF 4 h and NF 20 h treated samples were divided by the level of expression determined for the control samples, and the resulting ratios were compared to those obtained from the corresponding data derived from the RNA-seq experiments (from Table S2). The resulting plots (Figure S3) reveal some variability between the RNA-seq and RT-qPCR results, but no conflicting discrepancy.

**Figure 3:**
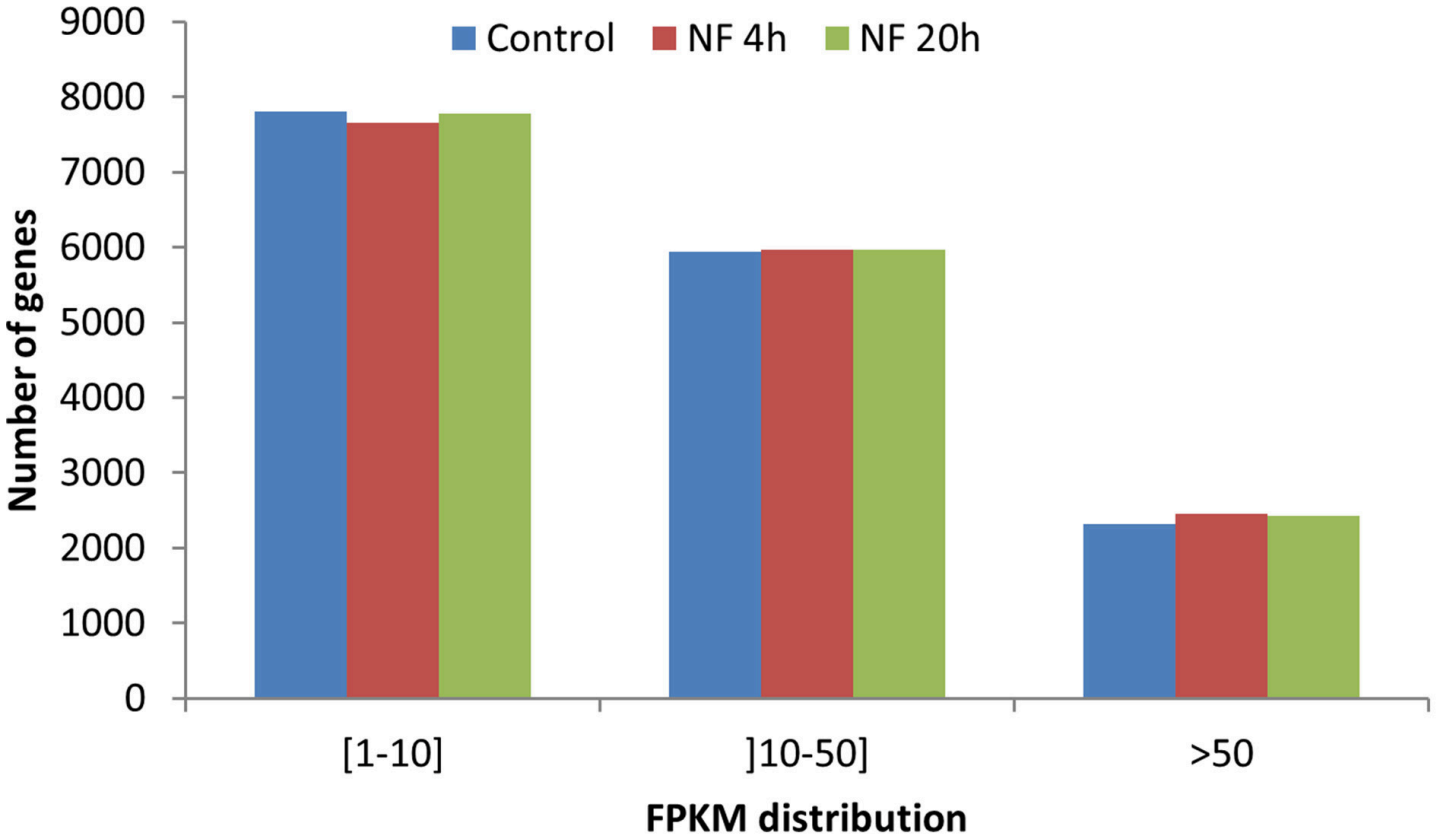
**Distribution of genes based on expression level in FPKM**. Three classes have been defined from weakly expressed [(1–10) FPKM] to highly expressed (>50 FPKM).

To describe the *M. truncatula* root hair transcriptome and to explore the relative expression levels of the different genes, the expression level of each gene was calculated in FPKM (Fragments Per Kilobase of exons per Million fragments mapped) values, from alignment of the paired-end reads to the improved *M. truncatula* genome release (Tang et al., 2014). For these analyses, only the longest transcript isoform has been considered. The genes with a FPKM higher than or equal to 1 were considered as expressed in root hairs. Sorting these genes into three classes of expression level (1–10, 10–50, and >50 FPKM) resulted in a similar distribution for the three treatments (Figure 3). In absence of NF treatment, 16,069 transcripts were identified as expressed in root hairs (Table S2), representing 31.5% of the predicted genes in the Mt4.0v1 genome assembly. The total number of expressed genes was only slightly affected by the NF treatments (16,077 and 16,170 for NF 4 h and NF 20 h, respectively; Table S2). Compiling the data from the three different treatments leads to a total of 16,810 non-redundant genes identified as expressed in *M. truncatula* root hairs in at least one of the tested conditions (Table S2).

### Impact of the NF treatments on gene expression

**Figure 4:**
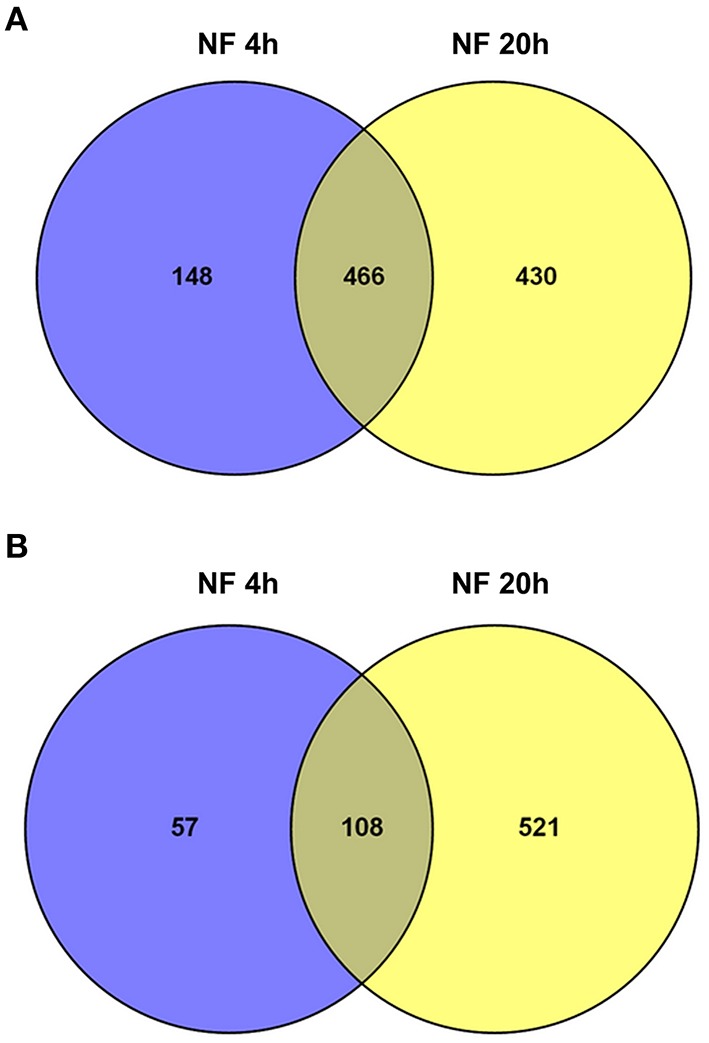
**Overview of significantly regulated genes identified in *Medicago truncatula* root hairs after Nod factor treatment**. Venn diagram summarizing the number of differentially expressed genes in the two time points following NF treatment, and their respective overlaps. **(A)** Up-regulated genes (6.1% of the genes expressed in root hairs). **(B)** Down-regulated genes (3.9%).

Transcriptional reprogramming in response to the NF 4 h and NF 20 h treatments was studied by assessing statistically significant differential gene expression between conditions, using EdgeR tool (Robinson et al., 2010). About 10% of the genes expressed in *M. truncatula* root hairs were thereby identified as significantly responding to NF, either being up- or down-regulated (with an adjusted *p* < 0.05; Table S3) 4 h and/or 20 h after the onset of the NF treatments. When compared with the expression data observed in the control root hairs, about 779 genes (614 up-regulated and 165 down-regulated) responded to the NF 4 h treatment, and 1525 genes (896 up-regulated and 629 down-regulated) to the NF 20 h treatment (Table S3). Among the genes identified as significantly responding to the 4 or 20 h NF treatments, 184 were specifically sensitive to the shorter treatment, 128 displaying up-regulation and 56 down-regulation, and 867 were specifically sensitive to the longer treatment, 349 displaying up-regulation and 518 down-regulation (Table S3). Thus, amongst the genes identified as responding to the NF 4 h treatment, a large majority (about 80%) displayed up-regulation. Amongst the genes identified as responsive to the NF 20 h treatment, up-regulation was again the more frequent case, but the corresponding percentage was then lower (60%). The Venn diagrams shown in Figure 4 indicate that about 45% of the genes were significantly up-regulated at both time points, 4 and 20 h, and that there is less overlap (ca. 16%) for the down-regulated genes.

**Figure 5:**
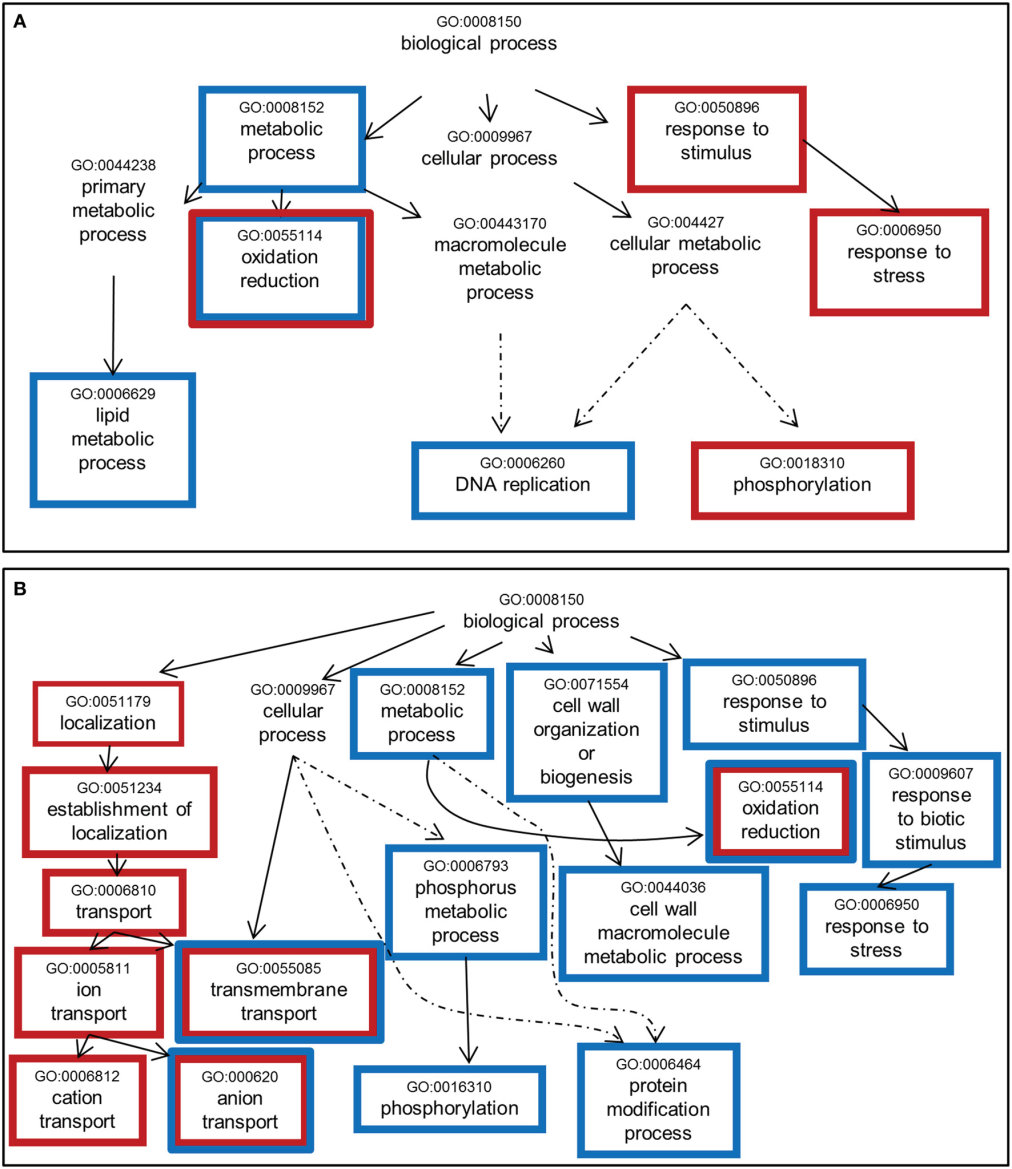
**Significantly enriched GO terms among genes whose expression is sensitive to Nod factor treatment. (A)** Up-regulated genes. **(B)** Down-regulated genes. One thousand seventy genes were annotated using agriGO toolkit (http://bioinfo.cau.edu.cn/agriGO/index.php). GO terms identified with an adjusted *p* < 0.05 are enclosed into boxes. Red and blue boxes refer to the changes in expression levels induced by the NF 4 h and NF 20 h treatments, respectively. Dashed arrow indicates that all the GO levels have not been represented.

A Single Enrichment Analysis (SEA) of GO terms (Du et al., 2010) within the list of NF-responsive genes was performed (Table S4). Considering the “biological process” category (Figure 5), we noticed some common enriched terms in response to NF 4 h and NF 20 h treatments such as “oxidoreduction,” “transmembrane transport,” and “anion/inorganic anion transport.” It is also worth to note that for both treatments (NF 4 h and NF 20 h), the analysis of the down-regulated gene list gave rise to about twice more significantly enriched GO terms than the up-regulated gene list. Over-represented semantic terms in the description of differentially expressed genes list was generated using the Gene Cloud software (Krouk et al., 2015). This analysis also identified the words “transporters” (or transport, uniport, symport) and “redox” (or oxydation, reduction, oxydoreductase) as amongst the enriched terms, especially in the description list of down-regulated genes at time 4 h with respect to the terms related to membrane transport (Figure S4).

### Plasma membrane transport systems involved in macro-nutrients uptake

**Figure 6:**
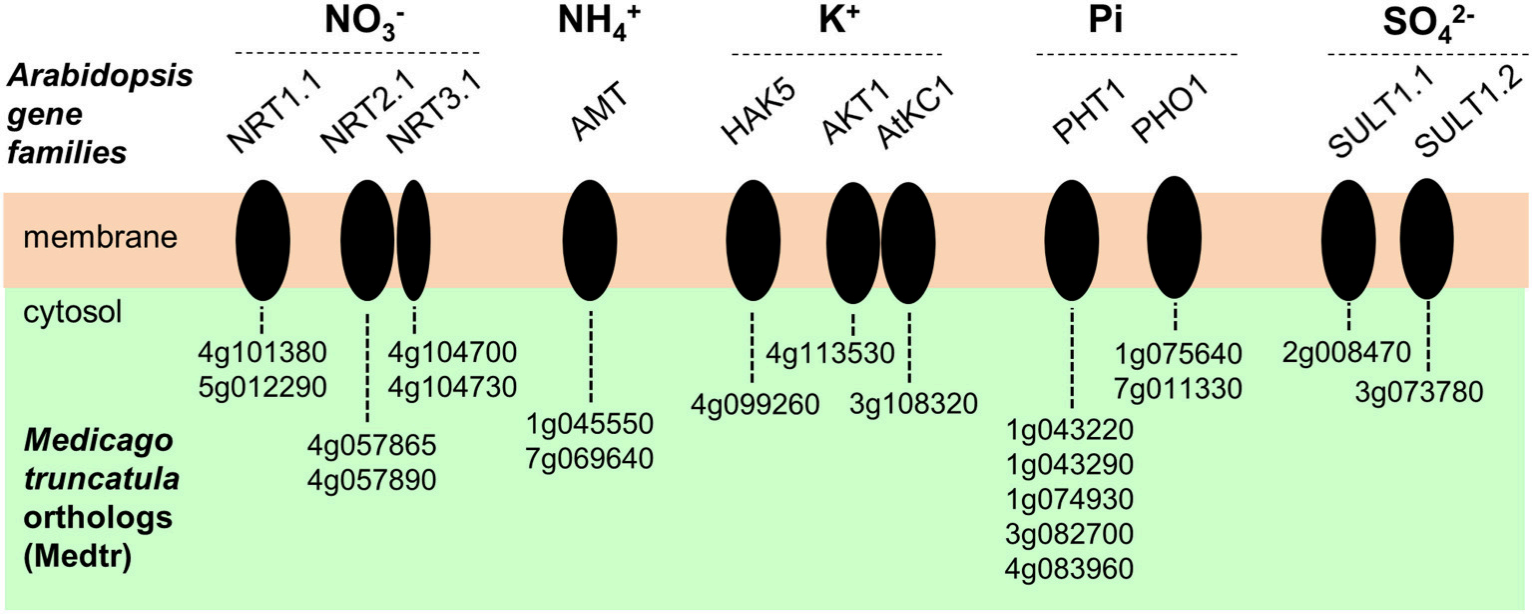
**Plasma membrane transport systems likely to play a role in transport of macronutrient ions in *Medicago truncatula* root hairs**. Candidate genes potentially involved in nitrate (${\text{NO}}_{3}^{-}$), ammonium (${\text{NH}}_{4}^{+}$), orthophosphate (Pi), potassium (K^+^) or sulfate (${\text{SO}}_{4}^{2-}$) transport are represented. The corresponding acronyms in Arabidopsis are provided. NRT, NitRate Transporter. AMT, AMmonium Transporter. HAK, High Affinity K^+^ transporter. AKT, Arabidopsis K^+^ transport system (from the Shaker channel family). AtKC1, *Arabidopsis thaliana* K^+^ channel 1 (member from the Shaker channel family). PHO1, PHOsphate 1. PHT1, PHosphate Transporter 1. SULT, SULfate Transporter.

Many homologs of genes identified in *Arabidopsis thaliana* or other species as playing a role in membrane transport of macronutrient ions were found to be expressed in *M. truncatula* root hairs. Candidate genes potentially involved in nitrate (${\text{NO}}_{3}^{-}$), ammonium (${\text{NH}}_{4}^{+}$), orthophosphate (Pi), sulfate (${\text{SO}}_{4}^{2-}$), or potassium (K^+^) transport are indicated in Figure 6. The *A. thaliana*–*M. truncatula* phylogenetic relationships of the corresponding gene families or subfamilies, named NPF (Nitrate transporter 1/Peptide transporter Family), NRT2, and NRT3 for ${\text{NO}}_{3}^{-}$, AMT (ammonium transporter) for ${\text{NH}}_{4}^{+}$, PHO1, and PHT1 for Pi, SULTR for ${\text{SO}}_{4}^{2-}$, HAK/KUP/KT, AKT1 (Shaker K^+^ channel subfamily 1), and AtKC1 (Shaker subfamily 4) for K^+^ are shown in Figures S5–S12, respectively. The expression level of these genes in the three conditions (control, NF 4 h and NF 20 h) are provided in Table S5. Interestingly almost half of the genes, expressed in root hair and potentially involved in nutrition functions, are regulated by NF 4 h or 20 h after NF treatment (17 up- and 10 down-regulated; Table S5). This proportion is significantly higher than that observed at the whole transcriptome level (ca. 10%; see above), indicating that the expression of the macronutrient transport machinery is especially reprogrammed following NF perception.

### Ion channels and pumps likely to play a role in early electrical and calcium signaling following NF perception

**Figure 7:**
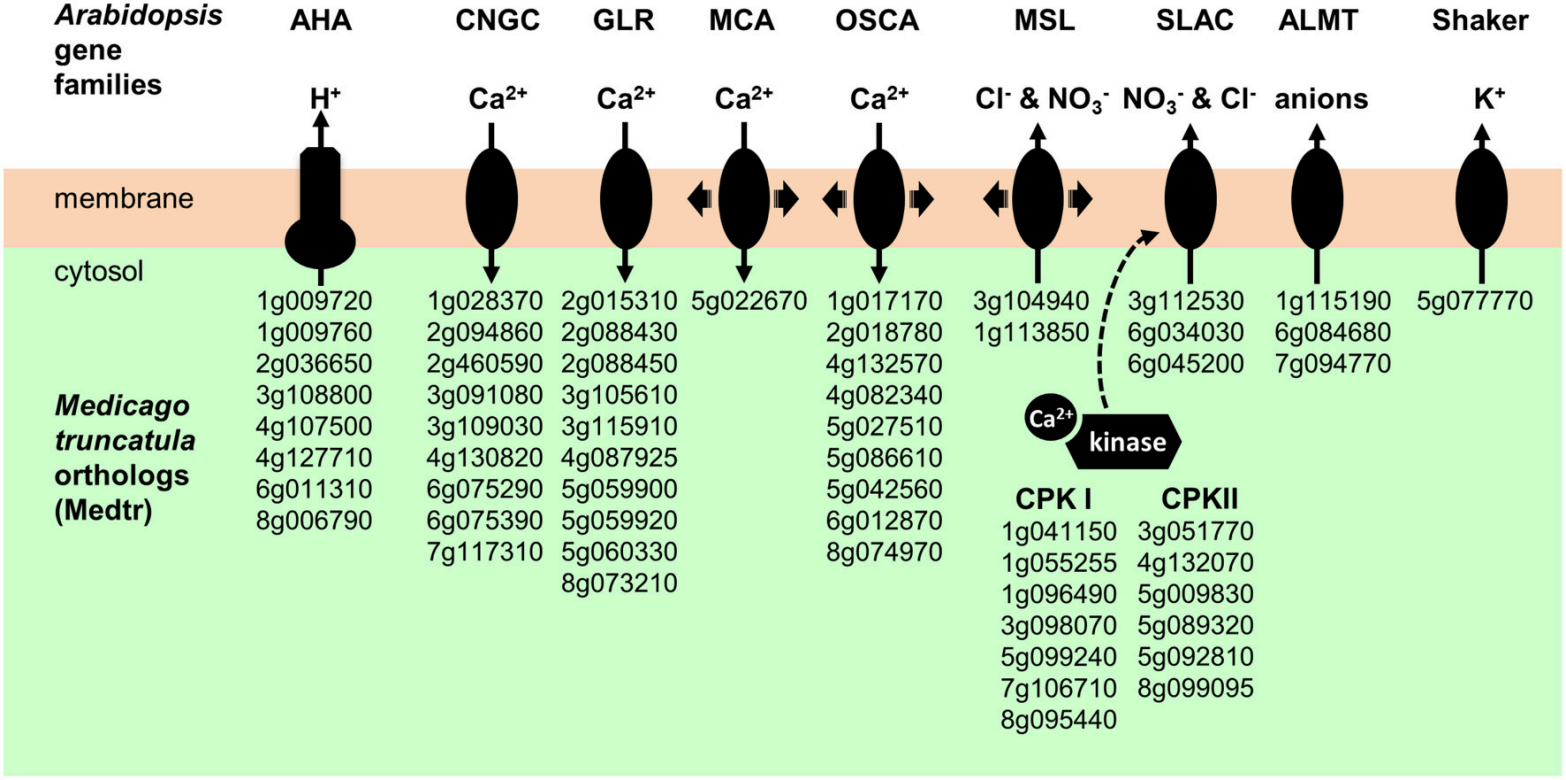
**Plasma membrane transport systems and regulatory kinases likely to play a role in early electrical and calcium signals triggered by NF perception**. AHA, Autoinhibited H^+^-ATPase. CNGC, Cyclic Nucleotide-Gated Channels. GLR, Glutamate Receptor-Like. MCA; Mid1-Complementing Activity. OSCA; Reduced hyperosmolarity-induced Ca^2+^ increase. MSL, Mechano-Sensitive MscS-Like channel. SLAC, Slow Anion Channel. ALMT, ALuminium-induced Malate Transporter. CPKI and CPKII, Calcium-dependant Protein Kinase I and II.

Early events triggered by NF perception are changes in ion fluxes through the root hair plasma membrane, resulting in electrical and calcium signals (Ehrhardt et al., 1992; Felle et al., 1998; Kurkdjian et al., 2000). The first events observed are an inhibition of H^+^ secretion and an activation of Ca^2+^ influx and Cl^−^ efflux, resulting in plasma membrane depolarization. Candidate proton pumps, calcium channels, anion channels, and potassium channels that could play a role in these early signaling events (see Discussion) are indicated in Figure 7.

In *M. truncatula* as well as in Arabidopsis and probably in all plant species, the plasma membrane proton pumps (H^+^-ATPase) are encoded by the AHA family (Falhof et al., 2016). Regarding the ion channel candidates, presently available information on gene families encoding such transport systems in plants, essentially gained in Arabidopsis, would lead to the following hypotheses: K^+^ channels could be encoded by the Shaker family, anion channels by the SLAC (Slow Anion Channels), ALMT (ALuminium-induced Malate Transporter), and/or MSL (mechano-sensitive MscS-like channels) families, and calcium-permeable channels by the CNGC (Cyclic Nucleotide-Gated Channels), GLR (Glutamate Receptor-Like), ANN (Annexin), MCA (Mid1-Complementing Activity), and CSC/OSCA (reduced hyperosmolarity-induced Ca^2+^ increase/Calcium permeable Stress-gated cation Channel). Genes belonging to these families and displaying expression in *M. truncatula* root hairs are listed in Table S5. Arabidopsis–Medicago phylogenetic relationships obtained for the Shaker, AHA, SLAC, ALMT, MSL, MCA, CSC/OSCA, CNGC, GLR, and ANN families are displayed in panel A of Figures S12–S19, respectively. The expression levels (in FPKM, obtained from the control root hair dataset) of genes belonging to these families are provided in panel B of these figures. It is noteworthy that among the 60 candidate genes selected as likely to encode membrane transport systems playing a role in early electrical and calcium NF signaling in root hairs following NF perception, only a few of them are found to be induced (10) or repressed (5) by the NF treatments (Table S5). Thus, these 60 candidate proteins are already present in root hairs before NF perception and their contribution (if any) to the signaling events triggered by NF perception would involve direct modifications of their transport activity rather than transcriptional responses.

### A repertoire of the root hair redox network

**Figure 8:**
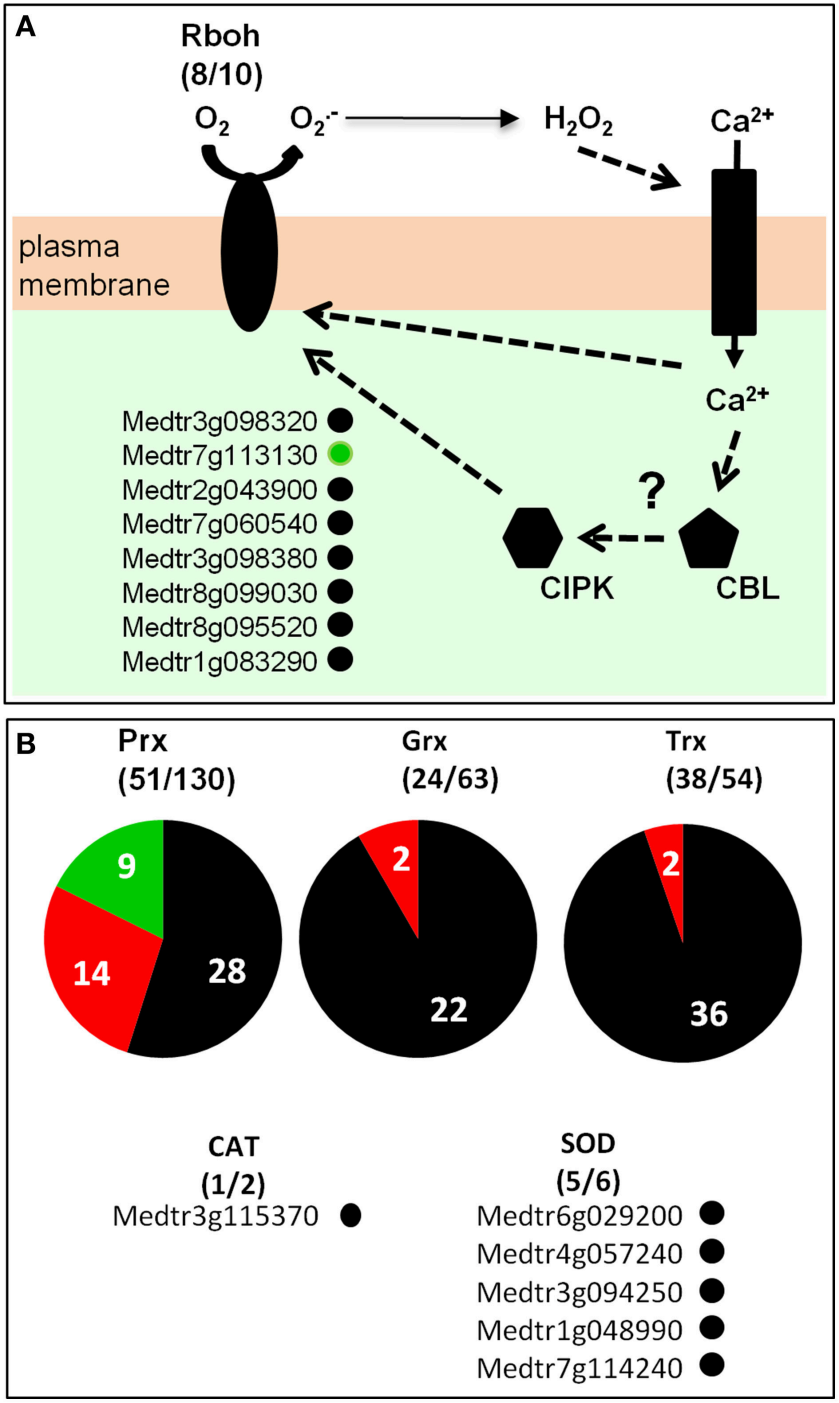
***Medicago truncatula* ROS network in root-hairs. (A)** RBOH (Respiratory Burst Oxidase Homolog) proteins catalyze the formation of superoxide anion, which is rapidly dismutated in hydrogen peroxide (H_2_O_2_) either spontaneously or by Superoxide Dismutase (SOD). H_2_O_2_ can regulate ion channels, which in turn modulate directly or indirectly RBOH activity possibly *via* calcineurin B-like-CBL-interacting protein kinase (CBL–CIPK)-mediated Ca^2+^ signaling. **(B)** Peroxidases (Prx), Glutaredoxins (Grx), Thioredoxins (Trx), catalases (CAT), and superoxide dismutases (SOD). For each gene family, the number of genes expressed in root hairs (over the total number of members of the corresponding family) is indicated. Dark gray, red, or green points or background: genes expressed in *M. truncatula* root hairs (FPKM value ≥ 1; see Table S6) that are insensitive, induced or repressed by NF treatment, respectively.

Genes involved in redox buffering or ROS synthesis/detoxification, according to the ROS network described by Mittler et al. (2004), were identified in the *M. truncatula* root hair transcriptome (Table S6; Figure 8). Among the well documented ROS producing systems, NADPH oxidases and peroxidases have been described as major sources of ${\text{O}}_{2}^{-}$ and H_2_O_2_, respectively, during plant development and in response to biotic and abiotic stresses (Marino et al., 2012; O'brien et al., 2012. Plant NADPH oxidases are named Respiratory Burst Oxidase Homologs (*Rbohs*). In *M. truncatula*, the *Rboh* family comprises 10 genes, among which eight are expressed in root hairs (Figure 8A; Figure S20). The most highly expressed gene is RbohD (FPKM > 200; Table S6). One member of this family, *RbohG*, is slightly down-regulated following 20 h NF treatment. In the *M. truncatula* genome (Mt4.0v1), 130 genes have been annotated as peroxidases (Figure 8B), among which 54 are expressed in the root hair transcriptome including 14 (9 up and 5 down) and 20 (12 up and 8 down) responding to the 4 h and 20 h NF treatments, respectively (Table S6).

Enzymes involved in ROS detoxification, namely superoxide dismutases (SODs) and catalases, are also expressed in *M. truncatula* root hairs. Indeed, five out of the six *SOD* genes present in the *M. truncatula* genome, together with one of the two *catalase* genes (Table S6), are found as expressed in the control root hair transcriptome.

### Legume specific genes expressed in root hairs

We investigated whether part of the *ca*. 16,000 *M. truncatula* genes identified as expressed in root hairs were specific to legume plant species. We defined legume-specific genes as protein-coding genes that have no predicted ortholog in any of the 25 non-legume species considered in our comparative analysis (Table S7). It should be noted that all the legume species included in this comparative analysis belong to the papillionid subfamily, which is the largest and most widespread legume family (Wojciechowski et al., 2004). We used the OrthoMCL software (Li et al., 2003; Fischer et al., 2011) to define groups of orthologous proteins among the predicted whole protein sets from 31 plant genomes (Table S7), including six legume genomes (three from the Phaseoloid clade, i.e., tropical or warm season legumes, and three from the Galegoid clade, i.e., temperate or cool season legumes). We identified 1176 *M. truncatula* genes expressed in root hairs and specific to legume species (Table S8). Among them, 138 genes were conserved in all the six considered legume species genomes. Comparison of the control, NF 4 h and NF 20 h transcriptomes identifies 26 and 74 legume-specific root hair expressed genes as responsive to the 4 and 20 h NF treatments, respectively, with a fold change >2 and an adjusted *p* < 0.05 (Table S8). Up-regulation was observed in a large majority (84%) of the genes identified as displaying sensitivity to the NF 4 h treatment, and in only 43% of the genes displaying sensitivity to the NF 20 h treatment.

## Discussion

### A valuable resource to investigate root hair functions in legumes

Polarized tip growth, nutrient uptake, and communication with microsymbionts are three interconnected aspects of legume root hair biology. For instance, curling of the root hair upon NF perception involves re-orientation of polarized tip growth (Esseling et al., 2003). Also, root hair elongation can be stimulated by reduction in nutrient ion availability (Giehl and Von Wiren, 2014), inoculation with rhizobacteria (Vacheron et al., 2013) or addition of isolated lipochitooligosaccharides (Dazzo et al., 1996).

The present study has been mainly focused on the roles of legume root hairs in mineral nutrition and communication with symbiotic rhizobia. It is well-known that proteins likely to play a role in these functions, like ion channels, can be expressed at very low levels. In the case of ion channels, low expression levels are likely to result from the fact that such proteins mediate very high transport rates. In this context, our objective has been to get a deep description of the root hair transcriptome by carrying out RNA-seq analyses since this methodology allows detection of very lowly expressed transcripts (Wang et al., 2009). RNA-seq analyses have previously been reported for several *M. truncatula* tissues, including roots (Boscari et al., 2013; Camps et al., 2015; Larrainzar et al., 2015) and nodules (Boscari et al., 2013; Cabeza R., et al., 2014; Cabeza R. A., et al., 2014; Roux et al., 2014; Avenhaus et al., 2015). To our knowledge, the present report is likely to provide the first RNA-seq transcriptome for *M. truncatula* root hairs and thereby the most exhaustive transcriptome available to date for this cell type. The effects of NF perception on root hair gene expression have been investigating at 4 and 20 h post-NF application.

We identify more than 16,000 genes expressed in *M. truncatula* root hairs (FPKM ≥1; Table S2), i.e., about 55 and 60% of the number of genes expressed in nodule tissues (ca. 30,000) or in whole root tissues (25,000–27,000; Boscari et al., 2013; Roux et al., 2014), respectively. Although, an FPKM cutoff value of one as a threshold to assess expression might be questionable, it is interesting to note that a similar proportion between the numbers of genes expressed in root hairs and in whole roots has been reported in Arabidopsis (Becker et al., 2014). About 10% of the ~16,000 genes expressed in root hairs were found to be significantly responsive to the NF treatments (787 and 1533 genes to the NF 4 h and NF 20 h treatments, respectively). In another legume model, *Glycine max*, Libault et al. (2010b) have investigated root hair responses to the symbiotic bacterium *Bradyrhizobium japonicum* using two complementary transcriptomic strategies (gene chip and RNAseq). This work led to the identification of 1973 genes that were differentially expressed in response to bacterial inoculation (Libault et al., 2010b). We also compared the present data with previous analyses of gene expression in *M. truncatula* roots (Czaja et al., 2012) or root hairs (Breakspear et al., 2014) carried out at 24 h post-NF application and performed using gene chip method (Table S10). From the 1050 and 849 genes identified as sensitive to NF treatment by Czaja et al. (2012) and Breakspear et al. (2014), respectively, about 35 and 42% display the same expression profile in our transcriptome analysis. Further, comparison with the single previous report on root hairs (Breakspear et al., 2014) is not straightforward since both the transcriptomic methods (gene chips vs. RNA-seq) and plant culture conditions were different. Indeed, Breakspear et al. (2014) used culture medium supplemented with aminoethoxyvinylglycine (AVG), an inhibitor of ethylene biosynthesis, probably leading to changes in hormone balance and root growth when compared with our experimental conditions. For instance, the two cytokinin response regulator genes *MtRR4* (*Medtr5g036480*) and *MtRR9* (*Medtr3g015490*) have been recently shown to be induced by NF treatment in presence of AVG, while only *MtRR9* is induced in absence of AVG (Van Zeijl et al., 2015). In agreement with this result, only *MtRR9* is identified as induced by NF in our transcriptome dataset (Table S2).

From the 1814 candidate genes proposed to play a role in polarized tip growth in Arabidopsis root hairs (Becker et al., 2014), we identified 1339 orthologous counterparts in the *M. truncatula* genome, among which 1089 are expressed in root hairs (Table S9). This suggests that the mechanisms underlying root hair tip growth are strongly conserved between *M. truncatula* and Arabidopsis. Providing further support to this hypothesis, 36 genes identified as crucial for root hair development and/or growth in Arabidopsis (Kwasniewski et al., 2013) are also found to be expressed in the present *M. truncatula* root hair transcriptomes. This includes genes involved in cell wall dynamics (e.g., expansin, pectate lyase, pectinesterase), in transcription regulation (bHLH) or in signaling (LRR-RLK, protein kinase; Table S10). It is also worth to note that *M. truncatula* root hairs expressed two homologs (*Medtr6g034030* and *Medtr6g045200*) of an Arabidopsis anion channel (AtSLAH3) known to play a role in pollen tube tip growth (Gutermuth et al., 2013). Altogether, these results provide evidence that the present transcriptome datasets constitute a valuable resource to further investigate major functions of root hair biology in *M. truncatula*.

### A panoply of nutrient ion transporters that includes unexpected systems

*In silico* mining of the transcriptome dataset in order to identify plasma membrane transport systems likely to play a role in plant mineral nutrition revealed candidate genes displaying significant expression levels in root hairs, whatever the nutrient ion we considered. The present results complete previous analyses establishing a genomic inventory of *M. truncatula* transporters (Benedito et al., 2010). Altogether, the results indicate that the *M. truncatula* root hair is a cell type particularly dedicated to plant mineral nutrition. Candidate genes likely to play a role in uptake from the soil solution of the macronutrients potassium, nitrate, ammonium, phosphate, and sulfate are listed in Figure 7; Table S5.

Two families of K^+^ transport systems, named Shaker and HAK/KUP/KT, have been identified in Arabidopsis as playing a major role in K^+^ uptake from the soil solution (Véry and Sentenac, 2003). The Shaker K^+^ channel family is strongly conserved in plants, as well as its regulatory network that comprises protein kinase from the CIPK (CBL-Interacting Protein Kinase) family (Cuellar et al., 2013; Sharma et al., 2013; Véry et al., 2014). In Arabidopsis, two Shaker inward K^+^ channel genes are expressed in root hairs, *AtAKT1* and *AtKC1*, giving rise to heteromeric K^+^ channels involved in passive “low affinity” K^+^ uptake from the soil solution (Sharma et al., 2013). The present transcriptome datasets indicate that only two inward Shaker channel genes are expressed in *M. truncatula* root hairs too, *Medtr4g113530* and *Medtr3g108320*, which are the closest relatives of the Arabidopsis *AtAKT1* and *AtKC1* (Figure S11). This suggests that these two genes encode Shaker subunits able to form heteromeric K^+^ channels like in Arabidopsis. It has also been shown that phosphorylation of AtAKT1 channel subunits plays a crucial activating role in regulation of inward K^+^ channel activity in roots (Sharma et al., 2013). The two calcineurin B-like calcium sensors CBL1 and CBL9 bind to the CBL-interacting protein kinase CIPK23, which then in turn phosphorylates AKT1 (Sharma et al., 2013). Interestingly, close homologs of these proteins (MtCIPK23: *Medtr2g049790*; MtCBL1: *Medtr3g091440*; MtCBL9: *Medtr4g099210*) are expressed in *M. truncatula* root hairs (Table S2). Regarding the HAK/KUP/KT transporter family, phylogenetic and transcriptome analyses (Figure S12) identify one gene, *Medtr4g099260*, that encodes a close homolog of the K^+^ transporter AtHAK5, shown to be a major contributor to active “high affinity” K^+^ uptake in Arabidopsis roots (Gierth et al., 2005). Thus, in our experimental conditions (0.7 mM external K^+^), *M. truncatula* root hairs seem to express both passive low affinity and active high affinity K^+^ uptake systems. Last, regarding K^+^ transport, *in silico* analyses reveal that *M. truncatula* root hairs express a transport system dedicated to K^+^ secretion into the external medium, *Medtr5g077770*, which is, within the *M. truncatula* Shaker family, the single ortholog of the two Arabidopsis outwardly rectifying K^+^ channels GORK and SKOR (Figure S12), the latter one being known as displaying activation by ROS (Garcia-Mata et al., 2010). Potential involvement of the Medtr5g077770 Shaker channel in establishment of nitrogen-fixing symbiosis will be discussed below.

Three families of membrane transport systems, named NPF (Nitrate transporter 1/Peptide transporter Family; Leran et al., 2014), NRT2 (Orsel et al., 2002), and NRT3 (Okamoto et al., 2006) in Arabidopsis, have been identified as playing a major role in nitrate uptake from the soil solution. The extensively studied AtNRT1.1/NPF6.3 “transceptor” is a dual affinity, bidirectional ${\text{NO}}_{3}^{-}$ transporter (Liu et al., 1999; Leran et al., 2013) as well as a ${\text{NO}}_{3}^{-}$ sensor mediating ${\text{NO}}_{3}^{-}$ regulated auxin transport, which thereby plays an important role in root development (Krouk et al., 2010a). In the root stele, AtNRT1.1/NPF6.3 is also involved in ${\text{NO}}_{3}^{-}$ secretion into the xylem sap toward the shoots (Leran et al., 2013), together with other NPF members (Lin et al., 2008; Taochy et al., 2015). A close homolog of AtNRT1.1/NPF6.3, *Medtr5g012290*, is the most highly expressed NPF member in *M. truncatula* root hair (Figure 7; Figure S5). Within the same NPF family, *Medtr5g093170* (MtNRT1.3/MtNPF6.8), which encodes a dual-affinity nitrate transporter similar to AtNRT1.1/NPF6.3 and demonstrated to play a role in control of root growth under N limitation (Morere-Le Paven et al., 2011), does not display expression in root hairs in our experimental conditions. NIP/LATD (*Medtr1g009200*), another NPF member from *M. truncatula*, is a high affinity ${\text{NO}}_{3}^{-}$ transporter (Bagchi et al., 2012) involved in lateral root and nodule development as well as primary root meristem maintenance (Bright et al., 2005). NIP/LATD is required for ROS homeostasis and cell elongation in roots (Zhang et al., 2014). We found this gene expressed in *M. truncatula* root hairs, although at a level ~6 times lower than that of *Medtr5g012290* (Figure S5B). *Medtr5g093170*/MtNRT1.3, which is a dual-affinity ${\text{NO}}_{3}^{-}$ transporter able to transport ABA and to mediate ${\text{NO}}_{3}^{-}$ inhibitory effects on primary root growth (Morere-Le Paven et al., 2011; Pellizzaro et al., 2014), was not expressed in root hairs in our experimental conditions.

In the NRT2 family, AtNRT2.1 is a ${\text{NO}}_{3}^{-}$ inducible-, high affinity ${\text{NO}}_{3}^{-}$ transporter (Li et al., 2007) and plays a key role in coordinating root development with ${\text{NO}}_{3}^{-}$ availability (Remans et al., 2006). *Medtr4g057890* and *Medtr4g057865*, close homologs to AtNRT2.1 (Pellizzaro et al., 2014), are expressed in *M. truncatula* root hairs (Figure 7; Figure S6). Within the Arabidopsis NRT3 family, AtNRT3.1 has been shown to physically interact with AtNRT2.1 to form functional heteromeric transport systems that provide the major contribution to high affinity ${\text{NO}}_{3}^{-}$ uptake from the soil (Yong et al., 2010). *Medtr4g104730*/MtNAR2.1 and *Medtr104700*/MtNAR2.2, the two Medicago homologs of AtNRT3.1, were by far most highly ${\text{NO}}_{3}^{-}$ transport-related genes expressed in Medicago root hairs in our study, suggesting a major role for these genes in ${\text{NO}}_{3}^{-}$ transport in *M. truncatula* root hairs.

Within the *M. truncatula* ammonium transporter family named AMT, the two members recently shown to have the highest levels of expression in roots as well as in shoots, *MtAMT1;1/Medtr1g045550* and *MtAMT2;1/Medtr7g069640* (Straub et al., 2014), are both expressed in root hairs (Figure 6; Figure S8). In contrast, the ammonium transporter *MtAMT2;3* (*Medtr8g074750*), which has been proposed to play a role in the uptake of ${\text{NH}}_{4}^{+}$ ions released by the fungus in plants engaged in arbuscular mycorrhizal (AM) symbiosis with *Glomus intraradices* (Breuillin-Sessoms et al., 2015), is not expressed in root hairs.

Evidence is available that root Pi uptake and transport is mainly mediated by transporters belonging to the PHT1 family (Nussaume et al., 2011). At least five members from the PHT1 family (out of 13) are expressed in *M. truncatula* root hairs (Figure 6; Table S5). This is in agreement with the hypothesis that root hairs play an essential role in plant Pi nutrition. It should also be noted that root hairs do not express *MtPT4*/*Medtr1g028600* and *MtPT8/Medtr5g068140*, two members from the PHT family shown to be induced upon AM symbiosis and then to contribute to the uptake of Pi ions released by the fungal membrane (Javot et al., 2011; Breuillin-Sessoms et al., 2015). Thus, it is tempting to speculate that, within large families of membrane transport systems such as the AMT and PHT families, some members have been specialized, maybe in terms of sensitivity to external pH, membrane polarization, or substrate concentration, in order to cope with the external conditions prevailing in either the soil solution or the apoplastic medium at the plant-microorganism interface.

*M. truncatula* root hairs can be assumed to also provide a major contribution to sulfate nutrition since they express *MtSULTR1.1/Medtr2g008470* and *MtSULTR1.2/Medtr3g073780*, which encode the closest homologs of the Arabidopsis *AtSult1;1* and *AtSult1;2* (Figure 7; Figure S10), the major contributors to high affinity sulfate uptake from the soil in Arabidopsis roots (Rouached et al., 2009). It is worth to note that while these genes are down-regulated in root hairs following NF treatment (Table S5), they are highly induced in mycorrhized *M. truncatula* roots as compared to non-mycorrhized roots whatever the growth medium S content (Gao et al., 2014; Wipf et al., 2014). In contrast, MtSULTR3.4b/*Medtr4g011970*, which has not been considered as expressed in control root hairs (FPKM < 1), is highly induced following NF treatment by almost 20 times. Thus, the route for sulfate nutrition appears different under normal condition or upon mycorrhiza or nitrogen-fixing symbiosis.

Two families of genes encoding membrane transporters, PHO1 and HKT, comprise members whose expression in *M. truncatula* root hairs could not be anticipated based on the present knowledge available in Arabidopsis or other plant species. The corresponding genes for the PHO1 family are *Medtr7g011330* and *Medtr1g075640* (Figure 6; Figure S9). Indeed, in Arabidopsis members from the PHO1 family (11 members), mainly PHO1 and PHO1; H1, are involved in Pi translocation into the xylem sap toward the shoots (Poirier et al., 1991; Hamburger et al., 2002; Stefanovic et al., 2007). Furthermore, direct evidence has been obtained that PHO1 is endowed with the capacity to mediate Pi secretion into the external (apoplasm) medium (Stefanovic et al., 2011; Arpat et al., 2012). It is thus tempting to speculate that the close homologs of PHO1 that are expressed in *M. truncatula* root hairs mediate secretion of Pi ions previously taken up by members from the PHT phosphate transporter family.

The HKT family comprises a single member in Arabidopsis, like in poplar and many dicots (Véry et al., 2014). Electrophysiological characterization of the Arabidopsis HKT, AtHKT1, the founder of plant HKT subfamily 1, has revealed a strong selectivity for Na^+^ against K^+^ and other cations, and no current rectification (Berthomieu et al., 2003). In other words, this transporter has the capacity to mediate Na^+^ uptake as well as secretion. It has been shown to be expressed in the plant vasculature, where it plays a role in desalinization of the ascending xylem sap in roots and recirculation of Na^+^ ions from shoots to roots via the phloem sap. Loss of function of this transporter results in increased plant sensitivity to salinity (Berthomieu et al., 2003). It is intriguing that the dicot *M. truncatula* possesses 4 HKT transporters, one of which displays expression in root hairs (*Medtr6g092840*; Figure 6; Figure S21). One may wonder whether such a number of HKT transporters contribute to *M. truncatula* tolerance to salt stress. This raises also the question of the actual roles of HKT transporters in root hairs and whether these roles involve Na^+^ uptake or secretion.

Finally, regarding nutrient ion transport in *M. truncatula* root hairs, it is worth to note that the equipment of this cell type in plasma membrane transporters or channels is likely to confer on it the capacity to secrete all the above considered nutrient ions, including Pi. Secretion of K^+^ and ${\text{NO}}_{3}^{-}$ into the external medium could play a role in the control of transmembrane electrical gradient and/or of internal water potential or pH upon abiotic stresses (Ivashikina et al., 2001; Segonzac et al., 2007). The physiological meaning of Pi secretion toward the external medium is however more difficult to decipher. A hypothesis would be that such equipment in secretion systems plays a direct role in the overall process allowing translocation of nutrient ions toward the inner tissues. This would involve polarized localization (or polarized activity) of the systems dedicated to secretion of nutrient ions, allowing root hairs to secrete ions into the root apoplasm toward neighboring cortical cells and not toward the external solution. Such an apoplastic step in the migration of ions toward the stele and xylem sap would make sense if diffusion through root hair plasmodesmata toward the cortical cells was a limiting step in the overall radial transport process.

### A repertoire of plasma membrane transport systems likely to play a role in early NF signaling

Early signaling events triggered by NF perception are changes in ion fluxes across the cell membrane, resulting in electrical and calcium signaling. The current model is that NF perception by root hair membrane receptors leads to an inhibition of the rate of H^+^ excretion by plasma membrane proton pumps and to an activation of calcium and anion channels, allowing Ca^2+^ influx and anion efflux. These events induce a strong depolarization of the cell membrane. This depolarization activates voltage-sensitive K^+^ channels, giving rise to an efflux of K^+^ ions, which results in membrane repolarization (Felle et al., 1996, 1998, 1999; Bouteau et al., 1999; Kurkdjian et al., 2000; for review, Oldroyd, 2013). At the molecular level, the present knowledge about the systems involved in these early NF signaling events is very low. Candidate genes listed in Figure 7 would however deserve to be tested by genetic and physiological analyses for a role in this signaling.

Plant plasma membrane proton pumps are encoded by the AHA gene family (Nguyen et al., 2015), which comprises five subfamilies in higher plants (Falhof et al., 2016). Eleven AHA genes are present in Arabidopsis and 13 in *M. truncatula*. Our data indicate that *M. truncatula* express five from these 13 genes in root hairs (Figure S13). Three genes, *Medtr4g127710, Medtr2g036650*, and *Medtr6g011310*, which have been recently named *MtAHA4, MtAHA5*, and *MtAHA6* (Nguyen et al., 2015), respectively, display much higher expression levels than the others. Interestingly, phosphoproteomic analyses of *M. truncatula* whole roots have revealed that 1 h treatment with NF leads to an increase in the phosphorylation status of the auto-inhibitory C-terminal domain of MtAHA5 pumps (Rose et al., 2012). Further, analyses have revealed that such a phosphorylation is likely to result in an increase in H^+^ pumping activity of these proteins (Nguyen et al., 2015). This could play a role in repolarization of the cell membrane following the initial NF-induced depolarization. On the other hand, this report does not provide information about the molecular mechanisms underpinning the initial inhibition of H^+^ secretion and membrane depolarization triggered by NF perception (Felle et al., 1996, 1998). Since the depolarization has been shown to occur very rapidly after NF perception, within a few minutes, it should essentially involve post-translational regulation. It is however worth to note that the present transcriptome data reveal a significant inhibition of *MtAHA5* expression (5 fold) as well as an increase in *MtAHA1* expression (80 fold) in response to NF treatment. *MtAHA1* had already been reported to be induced by both NFs and *S. meliloti* (Breakspear et al., 2014). *MtAHA1* has been shown to be specifically expressed in arbuscule-containing root cells and to be required for development of functional arbuscules (Krajinski et al., 2002, 2014; Wang et al., 2014). In our analyses, *MtAHA1* is not expressed in the control condition but strongly induced in the NF conditions (with a strongest induction at 4 h). Clearly, further genetic and biochemical analyses of the regulation and roles of the AHA pumps displaying expression in root hairs would be very useful. For instance, constitutively hyper-active AHA mutant pumps (genetically engineered based on the knowledge available in the Arabidopsis AHA family: Merlot et al., 2007) could be expressed in *M. truncatula* root hairs to assess the role of cell membrane depolarization in the early NF transduction pathway.

Regarding Ca^2+^, several gene families, amongst which the CNGC, GLR, ANN, MCA, and CSC/OSCA families, might encode channels involved in the initial Ca^2+^ influx and signals. The fact that, in animals, the CNGC, and GLR families play central roles in major signaling pathways has constituted a strong stimulus motivating extensive investigations of the roles of these systems in plants (Hedrich, 2012). Unfortunately, expression and functional characterization of plant CNGC and GLR has remained difficult or even unsuccessful in classical heterologous systems such as Xenopus oocytes, and the present knowledge about the transport activities of these systems is still quite unsatisfactory (Hedrich, 2012). It is thus very likely that progress in their functional characterization will essentially come from *in planta* investigations based on (electro) physiological and cell imaging analyses in mutant plants. Nonetheless, the fact that the plant CNGC and GLR families are relatively large, e.g., 20 CNGC and 20 GLR in Arabidopsis, 22 and 18 in *M. truncatula*, is likely to result in strong redundancy, at least in standard environmental conditions, and thus to render such analyses highly challenging. For instance, our present data identify 8 CNGC and 10 GLR (Table S5) genes as significantly expressed in *M. truncatula* root hairs. Interestingly however, for each of these two families, the expression levels of the genes expressed in this cell type vary within a very large range, by two orders of magnitude. It should also be mentioned that only few of them are regulated following 20 h NF treatment and none at the early time point. In this context, reverse genetics approaches to decipher the roles of these channels in early electrical signaling following NF treatment could be targeted to the genes displaying the highest levels of expression in root hairs. In plants, annexins are described as potential Ca^2+^-permeable channels involved in Ca^2+^ and ROS signaling (Laohavisit et al., 2010, 2012). In addition, annexins displayed phospholipid and F-actin binding activity suggesting a role in cytoskeleton rearrangements, membrane trafficking processes, and cell expansion. The Medicago genome contains 20 *Ann* genes, and about half of them are expressed in root hairs (Figure S19). Our data indicate that four from these genes are up-regulated by NF and one (*Medtr8g038210/MtAnn1*) is specifically expressed under NF treatment (Table S5) as it has already been reported (Breakspear et al., 2014). Interestingly, a close association between ANN1 expression and rhizobial infection has been previously shown (De Carvalho Niebel et al., 1998). *MtAnn1* protein displays a cytoplasmic localization but preferentially accumulates at the nuclear periphery of rhizobia-activated outer cortical cells, suggesting a role in the induction of Ca^2+^ spiking observed in rhizobial symbiosis (De Carvalho-Niebel et al., 2002).

With respect to the depolarizing efflux of anions, two candidate gene families can be thought about, the SLAC (Figure S14) and ALMT (Figure S15) families (Hedrich, 2012). Two members from the SLAC family, *Medtr6g045200* and *Medtr6g034030*, display high levels of expression in *M. truncatula* root hairs (Figure S14B) and they both are close homologs of the Arabidopsis AtSLAH3, which has been proposed to mediate anion efflux in Arabidopsis pollen tubes (Gutermuth et al., 2013). It is worthy to note that the genes encoding their regulatory kinases CPKI and II are also expressed in the root hair (Figure 7). In Arabidopsis, SLAC anion channels are activated by kinases from CPK (Ca^2+^-Dependent Protein Kinase) subfamilies I and II (Negi et al., 2008; Geiger et al., 2010). At least 13 CPK (7 from the CPK I subfamily and 6 from CPK II subfamily) potentially involved in SLAC channels activation are expressed in *M. truncatula* root hairs (Figure 7). Three ALMT genes are expressed in *M. truncatula* root hairs. In Arabidopsis, the ALMT family comprises 14 members, which are distributed into four clades, as also found in *M. truncatula* (Figure S15). ALMT are active either at the plasma membrane or at the vacuolar membrane (Barbier-Brygoo et al., 2011). In our study we found three ALMT genes expressed in root hairs, two of these, *Medtr7g094770* and *Medtr1g115190*, are up-regulated at 20 h post-NF application. They are homologous to AtALMT12 which is shown to encode a component of the R-type/QUAC anion current involved in guard cell signaling and requiring malate for activation (Meyer et al., 2010).

K^+^ channel activity encoded by the Shaker family is thought to dominate the plasma membrane conductance to K^+^ in most cell types in Arabidopsis (Lebaudy et al., 2007). Although this family is highly conserved in plants (Véry et al., 2014), the *M. truncatula* genome harbors a single outwardly rectifying Shaker K^+^ channel gene, *Medtr5g077770*, while two channels of this functional type have been identified in Arabidopsis, GORK, and SKOR. GORK is expressed in guard cells, where it mediates K^+^ efflux involved in stomatal closure (Hosy et al., 2003). It is also expressed in Arabidopsis root hairs, where its physiological role is still rather mysterious (Ivashikina et al., 2001). SKOR is expressed in root stele tissues where it contributes to K^+^ secretion into the xylem sap toward the shoots (Gaymard et al., 1998). Thus, the K^+^ channel encoded by *Medtr5g077770* is the single candidate from the Shaker family that can be proposed to play a role in cell membrane repolarization during the early electrical response to NF, besides being involved in stomatal movements and in K^+^ secretion into the xylem sap.

The presence of mechanosensors (MS) channels in the root hair plasma membrane, able to rapidly transduce mechanical forces into ion fluxes and electrical signals, is worth considering since adhesion of the microsymbiont and curling of root hair might elicit local mechanical constraints. In plants, MS channels are encoded by at least four small gene families, MscS-like (MSL; Haswell and Meyerowitz, 2006), Mid1-Complementing Activity (MCA; Kurusu et al., 2013), Piezo (Coste et al., 2012), and one larger family, CSC/OSCA (Hou et al., 2014; Yuan et al., 2014). Arabidopsis displays 10 MSL genes, which are distributed into two clades. Clade I comprises genes encoding proteins localized in endomembranes while clade II comprises proteins predicted or shown to be targeted to the plasma membrane (Haswell and Meyerowitz, 2006; Haswell et al., 2008). Two *MtMSL* of clade II are expressed in *M. truncatula* root hairs (Figure S16), *Medtr3g104940* and *Medtr1g113850*. They encode close relatives of the well-described Arabidopsis AtMSL10 and AtMSL8, which have been shown to be gated by membrane stretching. AtMSL10 is more permeable to anions (Cl^−^ and ${\text{NO}}_{3}^{-}$) than to cations (Haswell et al., 2008; Maksaev and Haswell, 2012). The pollen-specific AtMSL8 channel has been proposed to be involved in sensing of mechanical stress induced by hypoosmotic shock (Hamilton et al., 2015).

MCA channels have been initially identified in Arabidopsis and exhibit 10% identity to yeast Mid1 (Nakagawa et al., 2007). *M. truncatula*, like Arabidopsis, possesses two MCA genes, and one of them (*Medtr5g022670*) is expressed in root hairs (Figure S16). Available information on plant MCA provides support to a model wherein these proteins either are themselves MS Ca^2+^ channels or are closely associated with the activity of an MS calcium channel (Nakagawa et al., 2007; Yamanaka et al., 2010; Kurusu et al., 2012). It is noteworthy that the Arabidopsis MCA1 is likely to play a role in root mechanosensing and growth as suggested by a defect in root entry into hard agar of *mca1* mutant plants (Nakagawa et al., 2007; Yamanaka et al., 2010).

The present knowledge on the CSC/OSCA family (Hou et al., 2014; Yuan et al., 2014), which comprises 15 members in Arabidopsis and 13 members in *M. truncatula*, is still rather low. The two members identified in Arabidopsis, AtCSC1 (Hou et al., 2014), and AtOSAC1 (Yuan et al., 2014), have been shown to be activated upon hyperosmotic shocks and then to behave as poorly selective cation channels permeable to Ca^2+^. Among the 13 members of this family in *M. truncatula*, nine are expressed in root hairs (Figure S16; Table S5). Finally, the functional properties and physiological roles of Piezo family are still unknown in plant. The Piezo channel protein initially identified in mouse is an essential component of a cationic non-selective MS channel (Coste et al., 2012). *M. truncatula* has two homologs of the mouse Piezo, both expressed in root hairs (Figure S20) while only one homolog is present in Arabidopsis.

### A promising view of the ROS network in root-hairs

NADPH oxidases (RBOHs) have been shown to be important actors involved in ROS production in plants (Marino et al., 2012; O'brien et al., 2012. In *M. truncatula*, 8 out of its 10 *Rboh* genes are expressed in root hairs (Figure 8; Figure S21; Table S6), including the highly expressed gene (FPKM > 200) *MtRbohD*, which is the closest ortholog of Arabidopsis *AtRbohC* (Foreman et al., 2003), and *MtRbohF*, whose expression in root hairs was already reported (Marino et al., 2011). *MtRbohF* is the closest homolog of the two Arabidopsis NADPH oxidases *AtRbohH* and *AtRbohJ*. In Arabidopsis tip-growing pollen tubes, these two NADPH oxidases generate pulsating tip-localized H_2_O_2_ production that functions, possibly through Ca^2+^ channel activation, to maintain a steady tip-focused Ca^2+^ gradient during growth (Boisson-Dernier et al., 2013). Loss of function of *AtRbohC* in Arabidopsis (Foreman et al., 2003) and of a monocot-specific *Rboh* gene in maize plants (Nestler et al., 2014) has been reported to result in root-hairless phenotype. In *M. truncatula*, none of the investigations performed so far into the effects of loss-of-function mutations in *Rboh* genes (*RbohA*, Marino et al., 2011; *RbohC*, Zhang et al., 2014; *RbohE*, Belmondo et al., 2016) has revealed any root hair phenotype, suggesting strong gene redundancy. However, such analyses have not yet targeted the two genes, *MtRbohD* and *MtRbohF*, which are identified by our transcriptome data as the most highly expressed in root hairs.

In addition to NADPH oxidases, other enzymes, namely class III peroxidases (PRXs), are involved in H_2_O_2_ production during plant-pathogen interaction (Kadota et al., 2015). PRXs that are mainly localized in cell wall have been shown to play a role in cell wall remodeling (Francoz et al., 2015). The present transcriptome data identify more than 50 PRX genes as displaying expression in root hairs (Figure 8; Table S6). Sensitivity of such peroxidases to NF treatment in our experimental conditions is discussed later.

Transcription factors regulating the expression of peroxidases have been identified (Tsukagoshi et al., 2010; Sundaravelpandian et al., 2013). UPBEAT1, a basic helix-loop-helix (bHLH) root transcription factor, has been shown to regulate the expression of several peroxidases that fine-tune ROS accumulation between the zone of cell proliferation and cell elongation where differentiation begins (Tsukagoshi et al., 2010). A putative ortholog of UPBEAT1 is found in *M. truncatula* (*Medtr1g096530*), and our transcriptome data reveal expression of this gene in root hairs (Table S2). More recently, mutations in the Mediator subunit *MED25/PFT1* gene, which encodes part of a complex that docks transcription factors bound to enhancers with core promoter components, have been shown to result in compromised root hair development, due to altered expression of a set of H_2_O_2_-producing class III peroxidases (Sundaravelpandian et al., 2013). An *M. truncatula* PFT1 ortholog, *Medtr5g054680*, is expressed in root hairs (Table S2). With *UPBEAT*/*Medtr1g096530, MtPFT1/Medtr5g054680* may be another transcription factor playing a role in controlling peroxidase expression, and thus ROS levels, in *M. truncatula* root hairs.

Many genes encoding enzymes involved in ROS detoxification, namely superoxide dismutases (SODs) and catalases, are expressed in *M. truncatula* root hairs (Figure 8; Table S6). The present data indicate that almost all SOD genes are expressed in root hairs. This result, together with the fact that the NADPH oxidases, which synthesize ${\text{O}}_{2}^{-}$, are also almost all expressed in root hairs, suggest that the management of ${\text{O}}_{2}^{-}$ production and detoxification is of major importance and highly complex. Furthermore, it is now well established that ROS are important signals (Apel and Hirt, 2004) playing an essential role in Ca^2+^ channel activation in root hairs (Foreman et al., 2003; Takeda et al., 2008; Monshausen et al., 2009). Considering that the root hair is the entrance of the rhizobial symbiosis, these *M. truncatula* genes may be involved in steady state control of a tip-focused cytosolic Ca^2+^ gradient allowing fine tuning of calcium-regulated proteins, modification of the cytoskeleton, and localized vesicle exocytosis.

Finally, the presence in *M. truncatula* root hair transcriptome of sequences from at least 24 glutaredoxin (out of 63 in the genome) and 38 thioredoxin (out of 54) genes (Table S6) also provides evidence of the importance of redox control in root hair biology. Many Grxs and Trxs are present in plant genomes, but only a few of them have been shown to be expressed and characterized in root hairs among which the thioredoxin h5 (*At1g45145*) has been shown to be induced by pathogens and oxidative stress (Reichheld et al., 2002; Laloi et al., 2004).

### NF effects on expression of plasma membrane ion transport systems and ROS network

As discussed above, it makes sense that NADPH oxidases and most transport systems listed in Figure 7 as likely to play a role in the early electrical and calcium responses to NF are already significantly expressed in root hairs before NF arrival, *i.e*. that their expression in root hairs pre-exists to NF perception. However, enrichment analysis of GO terms indicates that, at a global level, NF treatments impact the transcription of genes belonging to categories related to membrane transport and redox activity (Figures 7, 8).

Various candidate genes encoding transport systems likely to contribute to nutrient ion uptake from the soil solution by root hairs, as hypothesized in Figure 6, are rapidly down-regulated at the transcriptional level upon NF perception (Table S5). For instance, this is the case for the two high affinity sulfate transporter genes SULTR1;1 (*Medtr2g008470*) and SULTR1;2 (*Medtr3g073780*) as indicated above, and of the high affinity phosphate transporter genes MtPT1 (*Medtr1g043220*) and MtPT6 (*Medtr3g082700*). Such results suggest that the root hair rapidly reduces its contribution to plant mineral nutrition a few hours after NF perception, at least regarding the uptake of nutrient ions such as phosphate and sulfate.

In contrast, *M. truncatula* homologs of Arabidopsis major representatives from the three families of ${\text{NO}}_{3}^{-}$ transport systems in Arabidopsis (NPF, NRT2, and NRT3) are not down-regulated upon NF treatments in *M. truncatula* root hairs, some of them being even up-regulated. This may suggest that nitrate fluxes are involved in plant response to NF. However, it is also worth to note that, in addition to their nitrate transport capacity, some Arabidopsis homologs of these *M. truncatula* transporters display other activities, leading to reprogramming of root architecture and modification of hormone fluxes or signaling events that may be of importance in the plant response to NF. For instance, both ${\text{NO}}_{3}^{-}$ transporters AtNRT1.1 and AtNRT2.1 are involved in ${\text{NO}}_{3}^{-}$ sensing and coordination of root development with ${\text{NO}}_{3}^{-}$ availability (Little et al., 2005; Remans et al., 2006; Krouk et al., 2010b). Interestingly, AtNRT1.1, the closest homolog in Arabidopsis of the *Medtr5g012290* transporter, also transports auxin (Krouk et al., 2010b), and control of auxin transport and accumulation is known to be involved in early nodule development (Mathesius et al., 1998; De Billy et al., 2001). Data on *Pseudomonas syringae/*Arabidopsis interactions suggest that AtNRT2.1 also participates in down-regulating biotic stress (salicylic acid-dependent) defense mechanisms through modifications in hormone pathways and sensitivity to the bacterial effector (Camanes et al., 2012). In addition, AtNRT2.1 was recently found to play a major role in control of root hydraulic activity (Li et al., 2016), a function which, in *M. truncatula*, may facilitate root hair development upon NF perception. In this context, it is worth noting that the two *M. truncatula* AtNRT2.1 homologs (*Medtr4g057865*/MtNRT2.2 and *Medtr4g057890*/MtNRT2.1) are highly expressed in root hairs and significantly stimulated by NF treatments (Figure S6). Since *MtNRT2.2* was found hardly detectable in total roots even by RT-qPCR (Pellizzaro et al., 2015), it is likely that *M. truncatula* root hairs are particularly enriched in MtNRT2.2, suggesting a specific role of this transporter in this cell type.

Seven out of eight *Rboh* genes expressed in root hairs display no significant transcriptional regulation upon NF perception (Figure 8; Table S6). One root hair expressed *Rboh, MtRbohG*, is weakly repressed (Figure 8; Table S6). On the other hand, several RBOH proteins, including MtRBOHD, have been found to be phosphorylated after NF application (Rose et al., 2012). In Arabidopsis, RBOHD is controlled by Ca^2+^ via direct binding to EF-hand motifs and phosphorylation by Ca^2+^-dependent protein kinases (Kadota et al., 2014, 2015; Li et al., 2014). Moreover, as mentioned above for AtAKT1 (Sharma et al., 2013), a direct interconnection between CBL–CIPK-mediated Ca^2+^ signaling and ROS signaling in plants provides evidence for a synergistic activation of the NADPH oxidase RBOHF. This activation would occur by direct Ca^2+^-binding to its EF-hands and Ca^2+^-induced phosphorylation by CBL1/9–CIPK26 complexes (Drerup et al., 2013). It is tempting to speculate that similar regulation mechanisms occur in *M. truncatula* root hairs too, giving rise to interactions between ROS and Ca^2+^ signaling pathways, since close homologs of Arabidopsis *CBL1/9* and *CIPK26* can be identified in the present transcriptome dataset (Table S2; Figure 8A). This would make sense with the fact that ROS signals and Ca^2+^ influx are very rapidly induced upon NF treatment, within a few seconds and then decreased (for review, see Felle et al., 1998; Puppo et al., 2013).

Except within the peroxidase family, only few genes involved in the ROS network as described by Mittler et al. (2004) are identified by the present data as sensitive to NF treatment (Table S6). The previous transcriptome data identified 10 “Rhizobially Induced Peroxidases” (*RIP1* to *RIP10*) genes, shown to be induced in root hairs by both *S. meliloti* and NF (Breakspear et al., 2014). One of these, *MtRip1*, was shown to be induced by H_2_O_2_ (Ramu et al., 2002). Interestingly, the expression patterns of these genes overlap with the sites of production of superoxide in infected root hairs, in nodules and roots (Chen et al., 2015). The present data (Table S6) reveal a set of 16 peroxidases genes displaying up-regulation upon NF perception, amongst which are 8 of the 10 previously identified RIP genes (Breakspear et al., 2014). All 16 of these peroxidases have a predicted secretion signal peptide, providing further support to the hypothesis of increased production of ROS in root hair apoplast during early NF signaling (Breakspear et al., 2014).

### Papilionoid legume specific genes expressed in *Medicago truncatula* root hairs: roles in symbiosis initiation?

We identified 1176 papilionoid legume-specific genes (LSGs) expressed in *M. truncatula* root hairs (Table S8). Previously, only 861 EST contigs had been identified as legume-specific in *M. truncatula* (Graham et al., 2004). Several classes of LSGs have been identified in *M. truncatula* including over 300 cysteine cluster proteins, 63 proline-rich proteins, and 21 glycine-rich proteins (Benedito et al., 2008). Within the present list of LSGs expressed in root-hairs (Table S8), about 4% (i.e., 46 genes), encode putative disease resistance proteins. Furthermore, most of the papilionoid legume-specific genes displaying high levels of expression in root hairs (FPKM > 50) are putatively involved in defense mechanisms. It is noteworthy that many of these papilionoid legume-specific defense genes, such as *Medtr5g088770* and *Medtr7g093820*, which encode a cysteine protease inhibitor (cystatin) and a disease resistance response protein, respectively, are down-regulated following NF treatment (Table S8). Such a down-regulation of defense genes might be required to allow the initiation of symbiosis. With respect to this hypothesis, GO analysis indicates that the semantic categories “response to stimulus” and “response to stress” are over-represented within the set of genes displaying up-regulation in response to the 4 h NF treatment (Figure 5; Table S4). In contrast, later on, 20 h after NF application, these categories are over-represented within the set of down-regulated genes. Altogether, these results provide further support to the hypothesis that the plant cannot straightforwardly welcome rhizobia as beneficial partners and engage in symbiosis without rapidly altering and adapting its immune/defense system. Also supporting this hypothesis are transcriptomic and phosphoproteomic studies that have revealed rapid induction of defense related genes and phosphorylation of proteins known to be involved in plant immune system (Libault et al., 2010a; Rose et al., 2012; Nguyen et al., 2015). Interestingly, about 50% of the legume specific disease resistance genes expressed in *M. truncatula* root hairs possess orthologs in all the legume genomes selected for the present analysis. It is thus tempting to assume that such genes contribute to central root hair functions common to all legumes, and that some of them play a role in rhizobial symbiosis.

Actin nucleation facilitated by the ARP2/3 complex plays a central role in plant cell shape development where the SCAR/WAVE complex is shown to be an essential upstream activator of ARP2/3 function in plants (Jorgens et al., 2010). The Suppressor of cyclic AMP receptor/WASP family verprolin homologous (SCAR/WAVE) complex is conserved from animals to plants and, generally, is composed of the five subunits SCAR/WAVE, PIR121 (p53-inducible protein-121), NAP125 (NCK-associated protein-125), BRICK, and ABI (Abl-interacting protein). Recently, several studies have shown the importance of these proteins in legumes (NAP1: NCK-ASSOCIATED PROTEIN, (Miyahara et al., 2010); PIR1: 121F-SPECIFIC P53 INDUCIBLE RNA, Yokota et al., 2009; LjSCARN, Qiu et al., 2015). *Medtr7g116710* gene, encoding an ABl-like protein (ABlL1) is early induced by NF (Table S10). This gene has also been reported to be induced by rhizobia (Breakspear et al., 2014). Interestingly, this gene has also been shown to be regulated by H_2_O_2_ (Andrio et al., 2013) and was found to be specific to papilionoid legumes in our OrthoMCL comparative genomics analysis (Table S3). It may be speculated that this ABIL1-like protein could have a function in linking SCAR/WAVE-dependent actin nucleation with ROS during the establishment of the rhizobial symbiosis.

## Conclusion

The present RNA-seq data provide the most exhaustive information available so far about gene expression in *M. truncatula* root hairs, before and after NF perception. These data have been analyzed by focusing mainly on genes likely to play a role in macronutrient ion transport across the root hair plasma membrane or in ROS signaling and early communication with rhizobia. These processes are of major importance for plant growth in natural ecosystems, when nutrient availability is limited. Hypotheses derived from the present transcriptome data identify candidate genes for reverse genetics investigation of a possible role in these processes and, more generally, provide a valuable resource for root hair systems biology.

## Materials and methods

### Plant growth and nod factor treatments

The whole experimental procedure was adapted from Sauviac et al. (2005). *M. truncatula* (ecotype Jemalong A17) seeds were scarified with sulfuric acid (99%) for 10 min and sterilized in 6% sodium hypochlorite solution for 3 min. After 3 h imbibition in sterile water, seed coats were removed and seeds were disposed on inverted 0.8% agar plates. The plates were kept for 48 h in the dark at 4°C. They were thereafter transferred at 21°C during 15 h for germination. Seedlings with a radicle long of about 2 cm were then transferred onto a sterile sheet (12 × 8.5 cm) of chromatography paper (Rogo-Sampaic, France) laid on solid Fahräeus medium in a Petri dish (12 × 12 cm, for 10 seedlings). Plantlets were grown in a growth chamber (70% humidity, 70 μE.m-2.s-1 light intensity) with a photoperiod of 16 h light (25°C) and 8 h dark (21°C) for 5 days in total before root hair isolation. Treatments with synthetic NF (Rasmussen et al., 2004) were performed either 4 or 20 h before root hair isolation (NF 4 h and NF 20 h treatments, respectively), by using 50 μl of 10 nM Nod factor solution per plant NodSm-IV(C16:2, Ac, S), gently deposited along of the root hair region (Figure 1A). Control roots were similarly treated with 50 μl of sterile water (Control Treatment). Root hairs were collected during the 7th hour of the light period on day 5. Two independent biological repeats were performed for all treatments, in each case with 100 plants.

### Root hairs isolation and RNA extraction

Root hairs were obtained from 4 cm-long root segments (Figure 1A) corresponding to the youngest part of the root hair zone. The root tip (ca. 0.5 cm) was excised to avoid contamination by root cap cells. Root hairs were isolated by freezing and vortexing the root segments in liquid nitrogen (Sauviac et al., 2005). About 60 mg of root hairs were obtained from 100 root segments.

RNA was extracted using the Qiagen RNeasy Plant Mini kit according to the supplier's instructions. DNA was removed using RNAse-free DNAse I (Qiagen) added directly onto the spin column. About 2.5–3 μg of RNA (measured using NanoDrop 1000 spectrophotometer, Thermo Scientific) were obtained from each root hair sample prepared from 100 plants. Total RNA were checked for their integrity on RNANano chip, using Agilent 2100 bioanalyzer (Agilent Technologies, Waldbroon, Germany).

### Transcriptome studies

RNA-seq experiments were carried out at the POPS-transcriptomic platform (IPS2-Saclay, France) using an IG-CNS Illumina Hiseq2000 machine to perform paired-end 100 bp sequencing on cDNA libraries performed by TruSeq_Stranded_mRNA_SamplePrep_Guide_15031047_D protocol (Illumina^®;^, California, USA). The 6 libraries have been sequenced in paired-end (PE) with a sizing of 260 bp and a read length of 100 bases. The multiplexing rate was three libraries per lane of Hiseq2000 machine (two lanes in total) to obtain around 50 million reads per sample.

### RNA-seq bioinformatic treatment and analysis

Each RNA-seq sample was subject to the same pipeline from trimming to count of transcript abundance as follows. The raw data (fastq) were trimmed to keep only bases with Phred Quality Score >20 and sequence read length >30 bases. Bowtie v2 was used to map reads to the *M. truncatula* transcriptome (Mt4.0v1 with—local option). The abundance of mRNAs was calculated by a local script which parses SAM files and counts only paired-end reads for which both reads map unambiguously to the same gene (i.e., reads mapping to multiple positions were discarded). According to these rules, around 73% of PE reads aligned to transcripts, in each RNA seq sample (Table S1). Differential gene expression across samples was analyzed using the EdgeR package (Version 2.4.6) in the statistical software “R” (Version 2.15.0). Raw read counts normalization was carried out with the EdgeR (v 2.4.6) software [with the Counts Per Million (CPM) function]. This method allows normalizing the data as a function of the size of the library. For each experimental treatment (control, NF 4 h, and NF 20 h) the libraries corresponding to the two replicates were normalized together, both libraries having the same normalization factor. For variability analyses, only genes with an average CPM >0 were considered. To control the false discovery rate, adjusted *p*-values were calculated using the Benjamini and Hochberg procedure (Storey and Tibshirani, 2003). Genes with an adjusted *p* < 0.05 were considered as being differentially expressed. In a complementary, we also wanted to explore the relative expression levels of the different genes with FPKM values. To achieve this, we aligned the quality checked reads to the *M. truncatula* annotated genome (Mt4.0v1) using TopHat (version tophat-2.0.10). Here again, reads mapping to multiple positions were discarded. As many as 85% of the read pairs aligned unambiguously to the genome. We then used Cufflinks (Trapnell et al., 2012) to estimate transcript abundance and generate FPKM values for all the corresponding genes (Table S2).

Assignment of Gene Ontology (GO) terms to differentially expressed genes was performed using agriGO (http://bioinfo.cau.edu.cn/agriGO/index.php) toolkit (Du et al., 2010). GO enrichment analysis of differentially expressed genes at 4 and 20 h following NF treatment was implemented using Singular Enrichment Analysis (SEA) with default parameters and *M. truncatula* genome locus (v4) as a background (Du et al., 2010). Briefly, a Fisher's exact test with a Benjamini-Yekutieli (FDR under dependency) adjustment method was used to classify the GO categories (Table S4). The significant GO terms were defined as having an adjusted *p* < 0.05.

### Orthomcl analyses

Predicted proteins from 31 fully sequenced plant genomes were collected from different sources (Table S7). This collection of plant proteomes included six legume species (*Medicago truncatula, Cicer arietinum* L., *Lotus japonicus, Glycine max, Phaseolus vulgaris*, and *Cajanus cajan*) as well as 23 other angiosperms (including monocots and dicots), one gymnosperm and a spikemoss (*Selaginella moellendorffii*) as an outgroup. An all against all comparison of all these 31 proteomes was performed using BLASTp (Altschul et al., 1997) with an *e*-value cutoff of 1e-5 and no SEG filter. The results of the all-against-all BLASTp analysis were fed to OrthoMCL v2.0 (Li et al., 2003; Fischer et al., 2011), which was run with a percentMatchCuttof of 50 and an inflation value of 1.5 for clustering granularity to create clusters of orthologous and in-paralogous plant proteins. Raw results of the OrthoMCL clustering analysis were parsed to identify clusters specific to legume species (clusters containing only legume genes). This list of legume-specific genes was crossed with the list of *M. truncatula* genes shown to be expressed in the root hairs. We then checked whether some of these legume-specific genes were conserved in all 6 legume species (included in our comparative proteome analysis).

### Phylogeny analyses

Except for the MSL family, polypeptide sequences were first aligned with Muscle (V3.8.31) and phylogenetic tree was generated with PhyML software (substitution model, LG matrix) using maximum-likelihood method and 1000 bootstrap replicates on Mobyle Pasteur website (http://mobyle.pasteur.fr/cgi-bin/portal.py#welcome). For MSL family, sequences were first aligned with Muscle using the conserved MscS domain as template to force the alignment around it (Jensen and Haswell, 2012). Phylogenetic trees were drawn with Dendroscope (http://ab.inf.uni-tuebingen.de/software/dendroscope/). Bootstrap values are indicated in gray at the corresponding nodes. The length of scale bar indicates the number of nucleotide substitutions per site.

### Data deposition

RNA-seq data from this article were deposited at Gene Expression Omnibus (http://www.ncbi.nlm.nih.gov/geo/, accession no. GSE67921) and at CATdb (http://urgv.evry.inra.fr/CATdb/; Project: NGS2013_04_POIL) according to the “Minimum Information About a Microarray Experiment” standards.

### RT-qPCR validation

For RT-qPCR validation, cDNA were obtained from root hair RNA extracted and treated as described above. The experiments were performed using three independent samples (biological replicates) for each treatment (control, NF 4 h, and NF 20 h), different from the samples used for the RNA-seq analyses and obtained in another laboratory than the one having prepared the latter RNA samples. The integrity of RNA was checked on agarose gel and RNA quantity and quality was assessed using NanoDrop 1000 spectrophotometer (Thermo Scientific). Total RNA (500 ng) was reverse-transcribed by Superscript III reverse transcriptase (Invitrogen, Paisley, UK) according to the manufacturer's instructions. Real-time RT-qPCR was carried out using the GoTaq master mix (Promega; www.promega.com) according to the manufacturer's instructions. Reactions were run on the Chromo4 Real-Time PCR Detection System (Bio-Rad; www.qiagen.com), and quantification was performed with the Opticon Monitor analysis software version 3.1 (Bio-Rad). Data were analyzed with RqPCRBase, an R package working in the R computing environment (Hilliou and Tran, 2013). The mRNA levels were normalized against two constitutively expressed endogenous genes (a38: *Medtr4g109650*; a39: *Medtr4g046877*). PCR for each biological replicate was performed in three technical replicates. For each reaction, 5 μl of 60-fold-diluted cDNA and 0.3 μM primers were used. The initial denaturing time was 10 min, followed by 40 cycles at 95°C for 10 s and 60°C for 1 min. Specificity of amplification was confirmed by observing a single peak in the dissociation curves at the end of the PCR procedure. The gene-specific primers used are listed in Table S11.

## Author contributions

ID, AD, MG, SB, VB carried out the experimental research. SC, SF, ER performed the chemo-enzymatic synthesis of NF. ID, AD, MG, IG, AB, AP, JMF, NP, HS evaluated the results, and contributed to writing the manuscript. MG, AB, SB, JCB, VB, ED, MD, VM, CF, CR, HR, YS, JT, BT contributed to RNA-seq analyses and biocomputing. ID, AD, AP, NP, HS wrote the paper. AP, JF, NP, HS conceived the project, participated in its design and coordination. All authors read and approved the final manuscript.

## Funding

This work was funded by an Agence Nationale de la Recherche (ANR) project (CAROLS—Channels and Reactive Oxygen species in Legume root hair: role in Symbiosis with Rhizobium—ANR-11-BSV7-010-02). This work was supported by the “Institut National de la Recherche Agronomique,” the “Centre National de la Recherche Scientifique,” the University of Nice Sophia Antipolis and the French Government (National Research Agency, ANR) through the LABEX SIGNALIFE program (ANR-11-LABX-0028-01). ID was supported by a post-doctoral fellowship from the ANR (CAROLS: ANR-11-BSV7-010-02). AD was supported by doctoral fellowship (PER, Prix d'Encouragement à la Recherche) from the “Province Sud de la Nouvelle-Calédonie.” SC, SF, ER are grateful to the Labex Arcane (ANR-11-LABX-0003-01) and the Carnot Polynat Institute for partial support of this study and the NMR and Mass Spectrometry Platforms of the ICMG (FR 2607).

### Conflict of interest statement

The authors declare that the research was conducted in the absence of any commercial or financial relationships that could be construed as a potential conflict of interest.
