# Supplementary material for: Nod Factor Effects on Root Hair-Specific Transcriptome of Medicago truncatula: Focus on Plasma Membrane Transport Systems and Reactive Oxygen Species Networks
Source: Front Plant Sci. 2016 Jun 7;7:794. doi: 10.3389/fpls.2016.00794 (PMC4894911; doi:10.3389/fpls.2016.00794)
Supplement: Supplementary file 12 [file Image1.PDF]

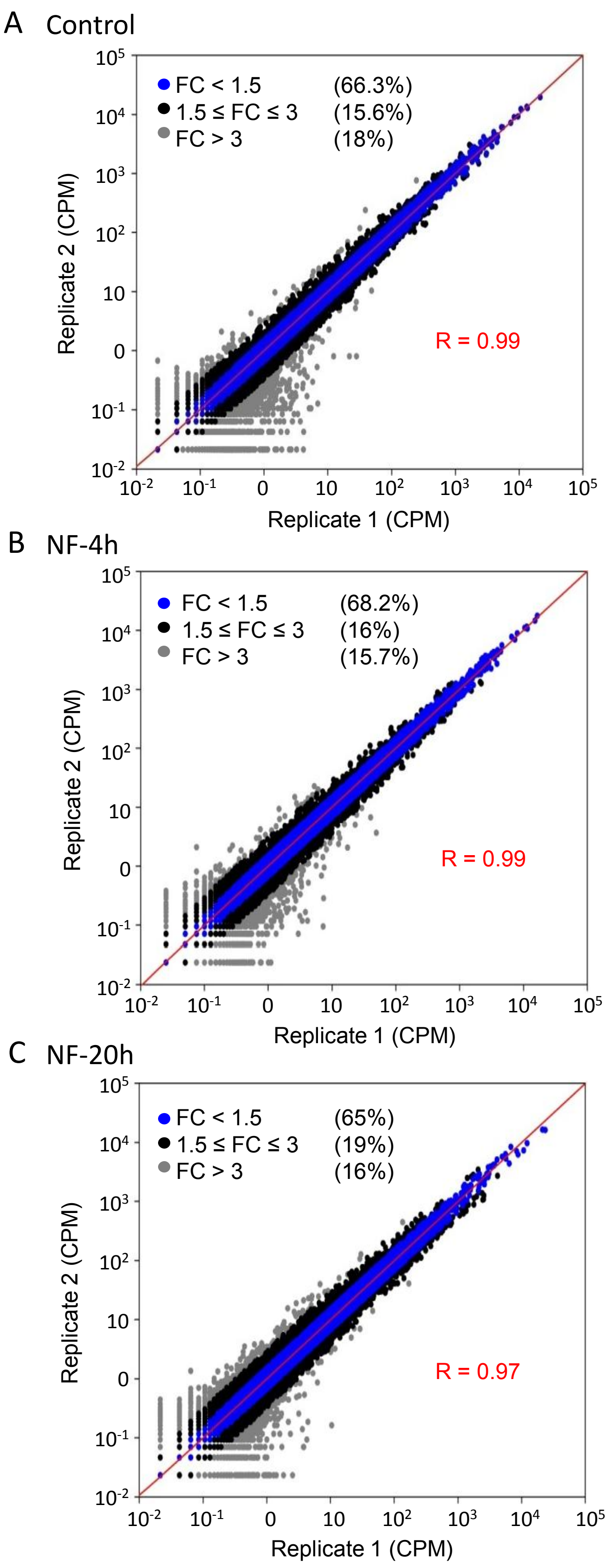


**Figure S1: Analysis of the variability between the two biological replicates of the control and NF treatments. (A)** Control treatment. **(B)** NF-4h treatment. **(C)** NF-20h treatment. The levels of transcript accumulation are provided in CPM (Counts Per Million of mapped paired reads) using a common logarithmic scale. The experimental points corresponding to genes with a fold change between the two replicates lower than 1.5, or between 1.5 and 3 or higher than 3 are spotted in blue, black or grey, respectively. The percentage of genes in each of these 3 categories is indicated in brackets. The linear regression line and the correlation coefficient R are provided in red on the graph.

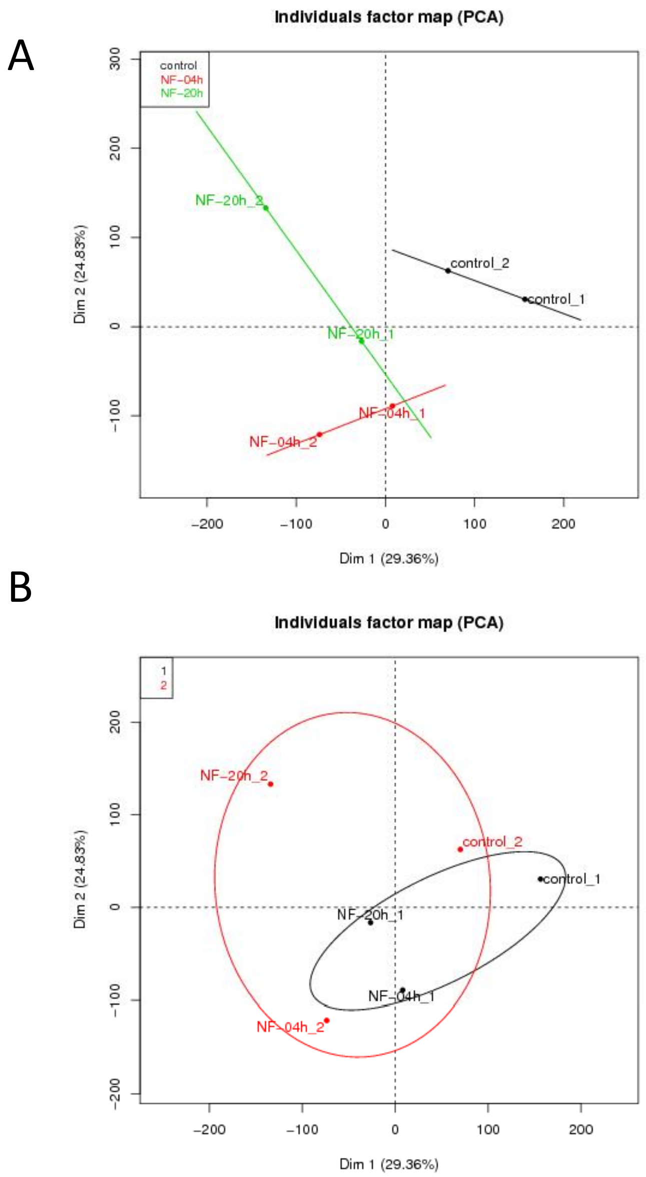


**Figure S2: Principal component analysis of RNAseq data.** Gene expression values from each biological repetition are projected onto the first two principal components (Dim1 and Dim2). **(A)** PCA of sample treatments before data normalization showing a clear separation between controls (black) and treated samples (NF-04h, red; NF-20h, green). **(B)** PCA of biological repetitions before normalization showing a homogeneous distribution meaning that there is no biological repetition bias. Lines indicate a confidence ellipse at 95%. Samples are colored according to the 2 biological replicates: replicate 1 (black), replicate 2 (red).

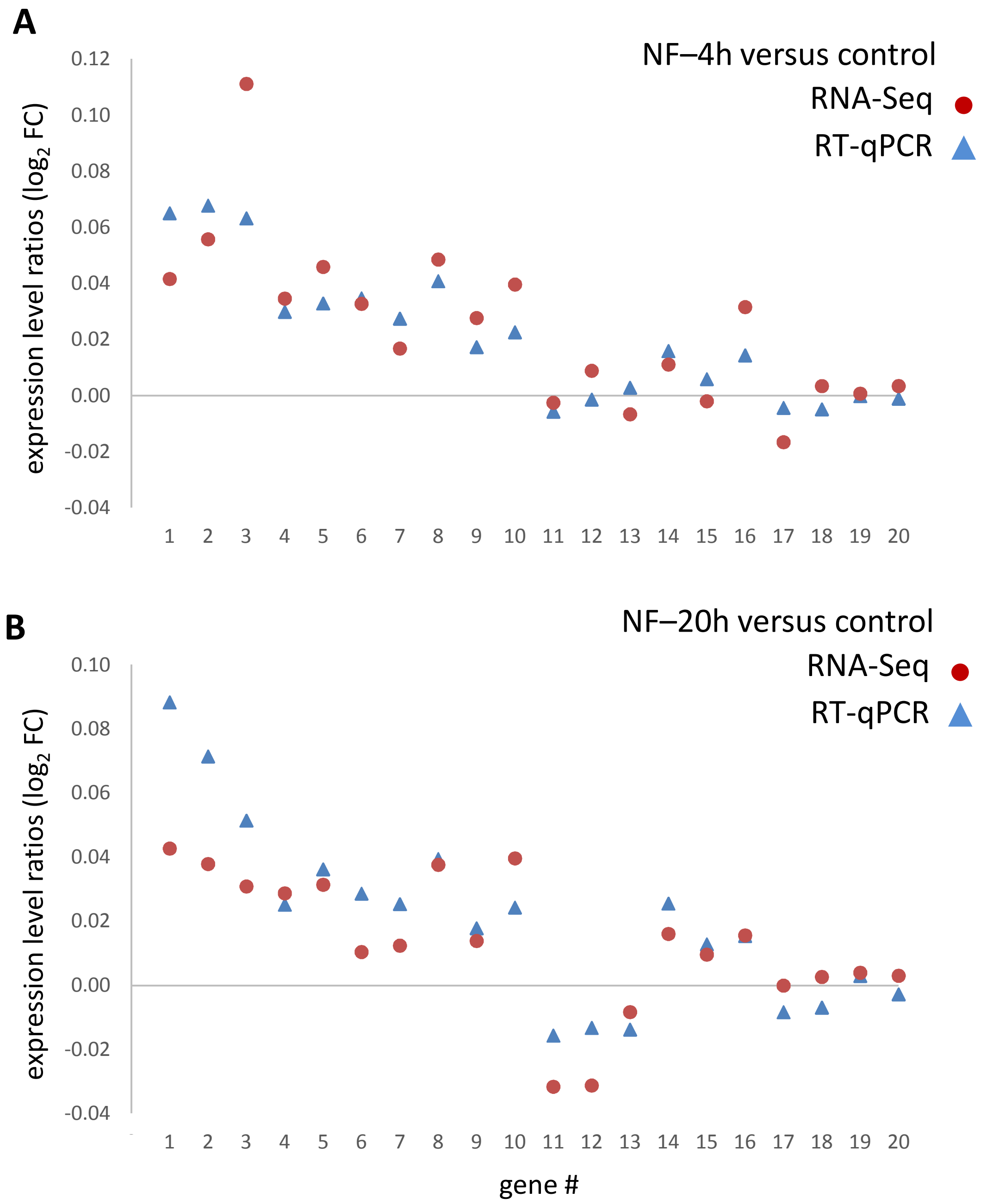


**Figure S3: Comparison of RNA–Seq and RT-qPCR data for a representative set of 20 genes.** X axis: the selected genes are numbered from 1 to 20. This selection (Supplemental table S11) comprises genes encoding receptors, pumps or channels differing in their levels of expression and representing different expression patterns in response to NF treatments. Y axes (note the logarithmic scale): relative fold changes in expression levels. For each gene from the selection and each set of data (RNA-Seq or RT-qPCR), the expression level in the NF-4h treatment (Panel A) or NF-20h treatment (Panel B) has been divided by the corresponding data in the control treatment. Red circles: ratios computed from the RNA-Seq datasets. Blue triangles: same ratios but computed from the RT–qPCR data. Selected genes (supplemental table S11): (1) Medtr5g011960 (Lipid transfer protein); (2) Medtr5g033490 (LysM-RLK); (3) Medtr4g081190 (ABC transporter B family protein); (4) Medtr8g020840 (GRAS family transcription factor); (5) Medtr4g129010 (tyrosine kinase family protein); (6) Medtr1g086550 (plant gibberellin 2-oxidase); (7) Medtr3g096500 (gibberellin 20-oxidase); (8) Medtr4g011970 (sulfate/bicarbonate/oxalate exchanger and transporter sat-1); (9) Medtr6g007697 (potassium transporter-like protein); (10) Medtr2g023890 (MAP kinase kinase kinase); (11) Medtr7g113130 (respiratory burst oxidase-like protein); (12) Medtr2g035190 (ABA-responsive protein); (13) Medtr3g069500 (peptide/nitrate transporter plant); (14) Medtr3g110405 (Serine/Threonine-kinase aurora-like protein); (15) Medtr3g105610 (glutamate receptor 3.2); (16) Medtr5g071560 (MAP kinase kinase kinase); (17) Medtr4g027040 (NF-X1-type zinc finger protein NFXL1); (18) Medtr3g104940 (mechanosensitive ion channel family protein); (19) Medtr7g06254 (auxin response factor 2); (20) Medtr5g077770 (potassium outward rectifying channel protein).

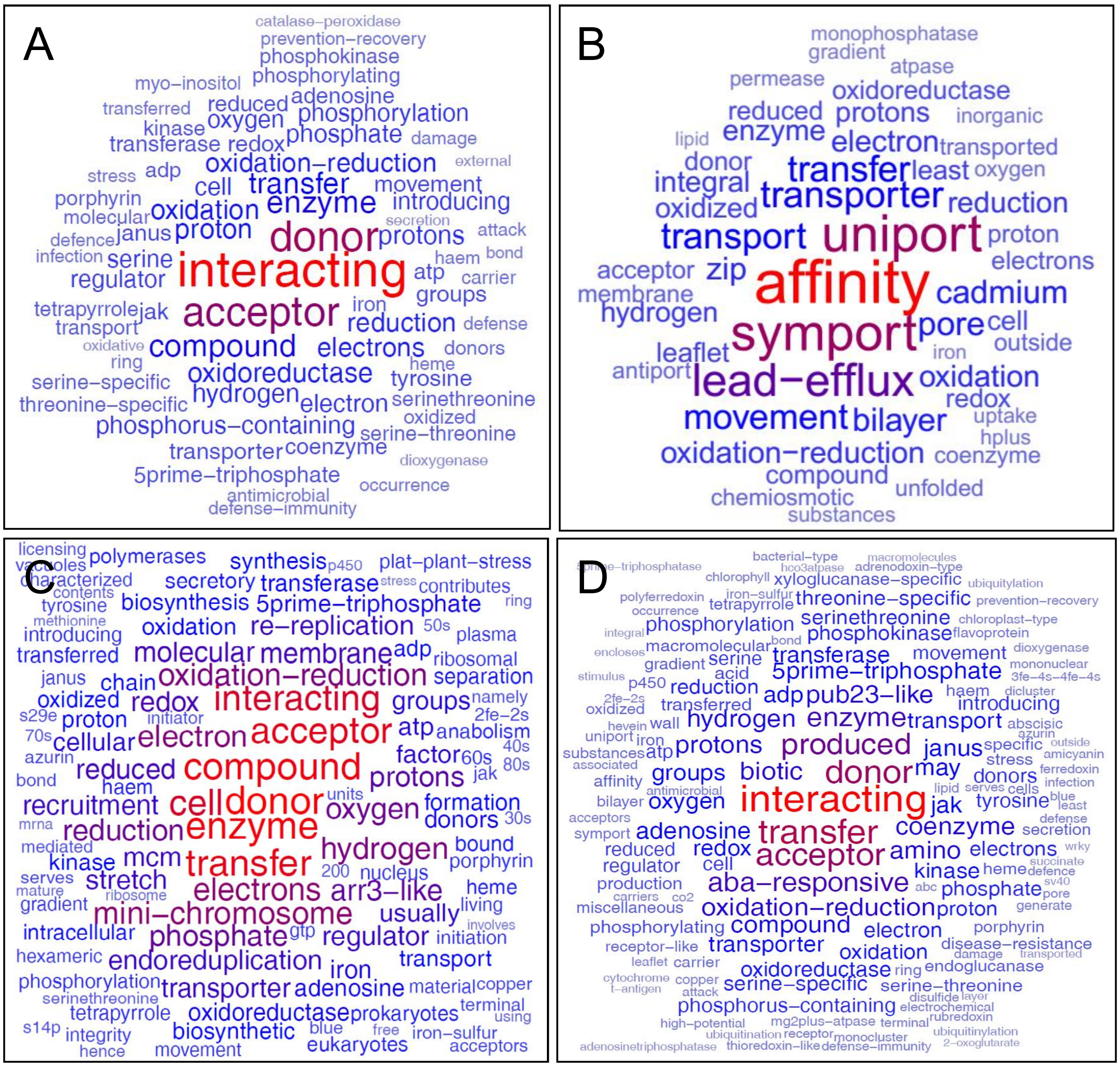


**Figure S4: GeneCloud – semantic terms over-represented in the description list of differentially expressed root hair genes following NF treatments.** The list of genes significantly induced (**A, C**) or repressed (**B, D**) by NF treatment at 4 h (**A, B**) and 20 h (**C, D**) were submitted to https://www.m2sb.org/ using the following parameters: “keep terms occurring in at least 3 genes”, “FDR threshold (α) 1‰”, “p.adjust method Bonferroni”).

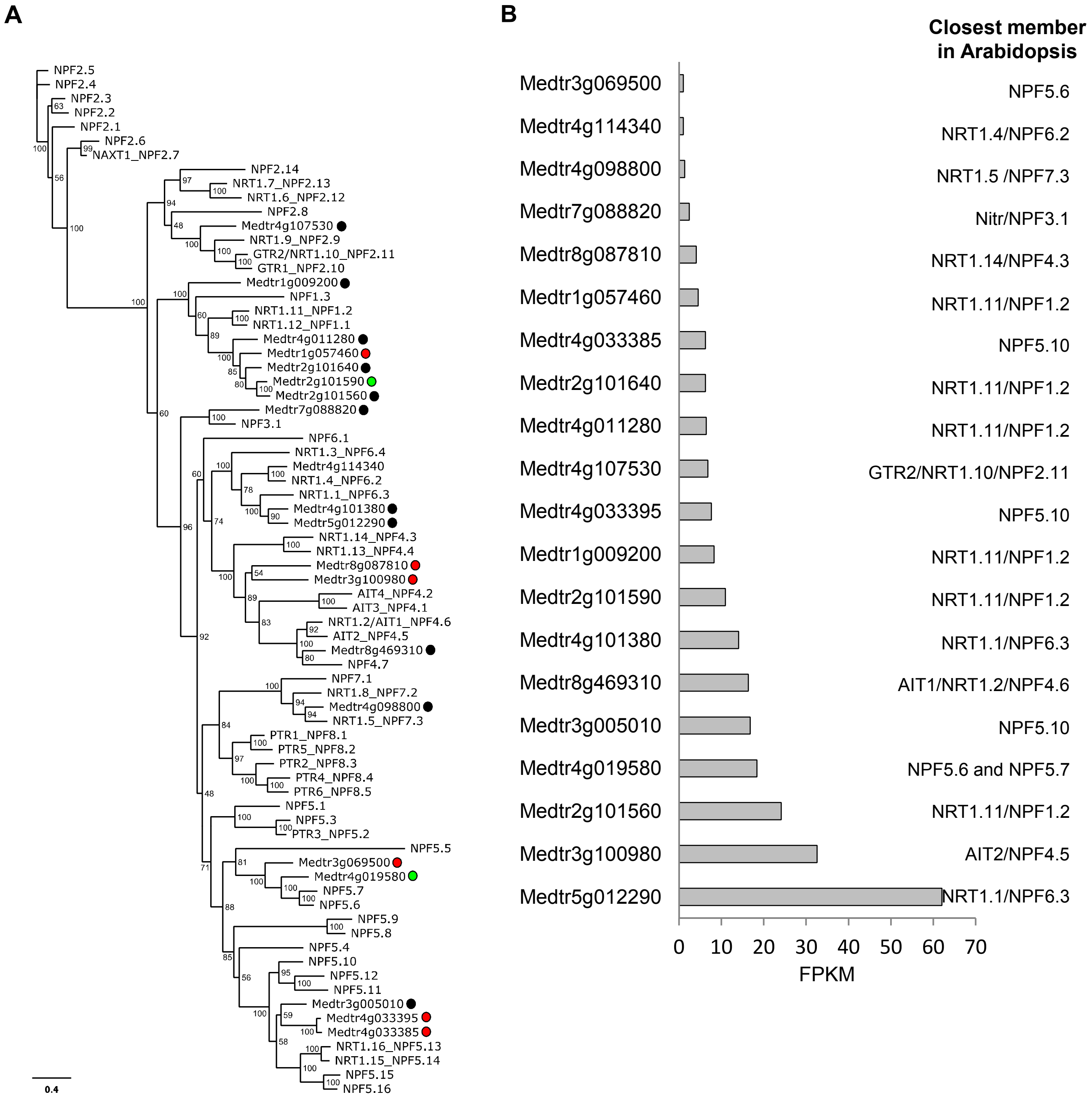


**Figure S5: Members of the Nitrate Transporter 1/Peptide Transporter family (NPF) expressed in *Medicago truncatula* root hairs.** (**A**) Phylogenetic relationship of *A. thaliana* and *M. truncatula* NPF proteins. When available, alternative names are provided. For the sake of clarity, *M. truncatula* NPF members whose genes are not expressed in root hairs (FPKM value < 1; see Table S2) were not included in this phylogenetic tree. Genes expressed in root hairs (FPKM value ≥ 1; see Table S2) are labeled with colored circles: red, green and black circles correspond to genes induced, repressed or not regulated by the NF treatments, respectively. (**B**) Relative expression (in FPKM) of *M. truncatula NPF* genes in control root hairs.

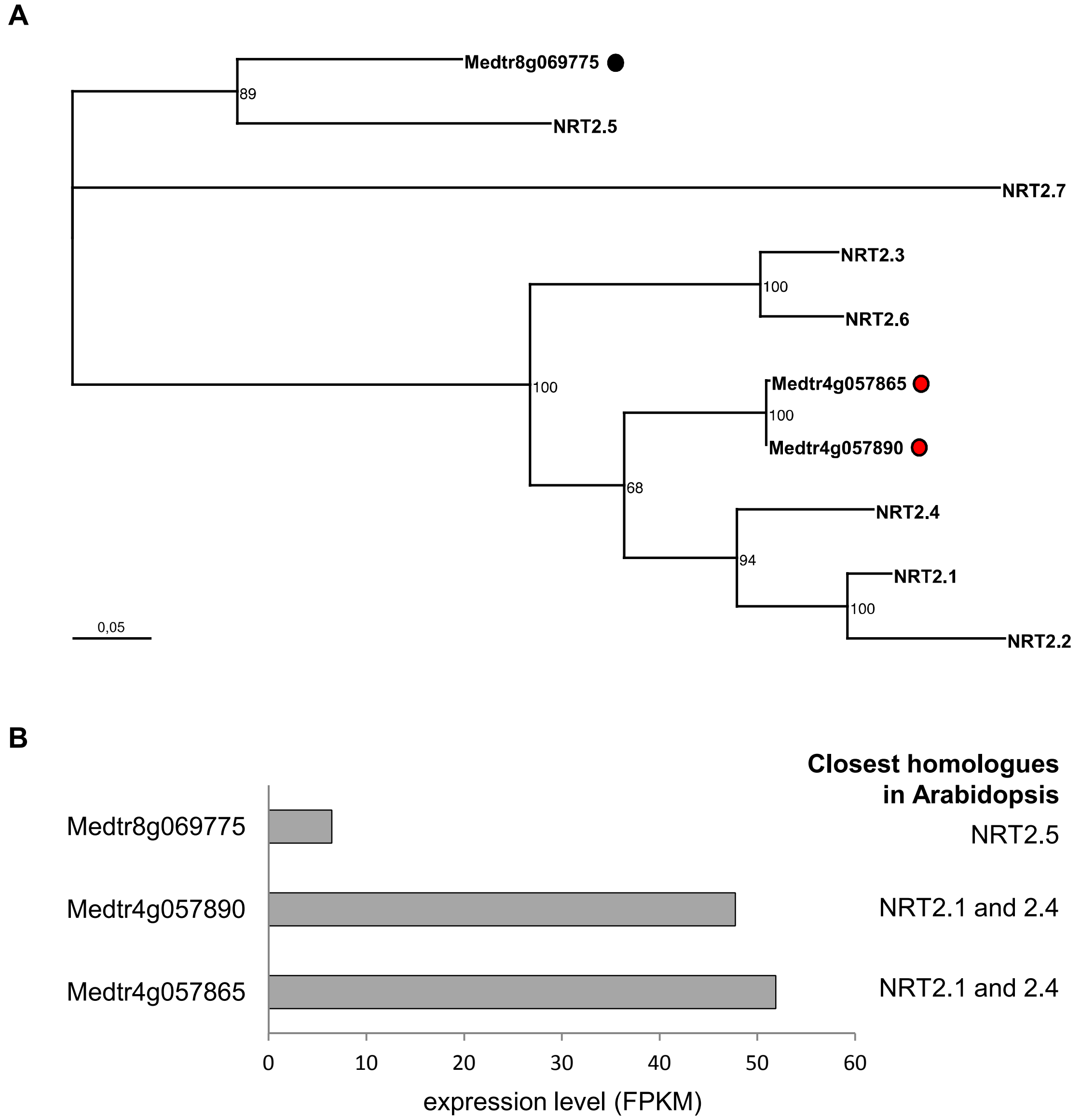


**Figure S6: The NRT2 nitrate transporter family in *Medicago truncatula* and its members displaying expression in root hairs.** (**A**) Phylogenetic relationship of *A. thaliana* and *M. truncatula* NRT2 proteins. Genes expressed in root hairs (FPKM value ≥ 1; see Table S2) are labeled with colored circles: red and black circles correspond to genes induced or not regulated by NF treatments, respectively. (**B**) Relative expression (in FPKM) of *M. truncatula NRT2* genes in control root hairs.

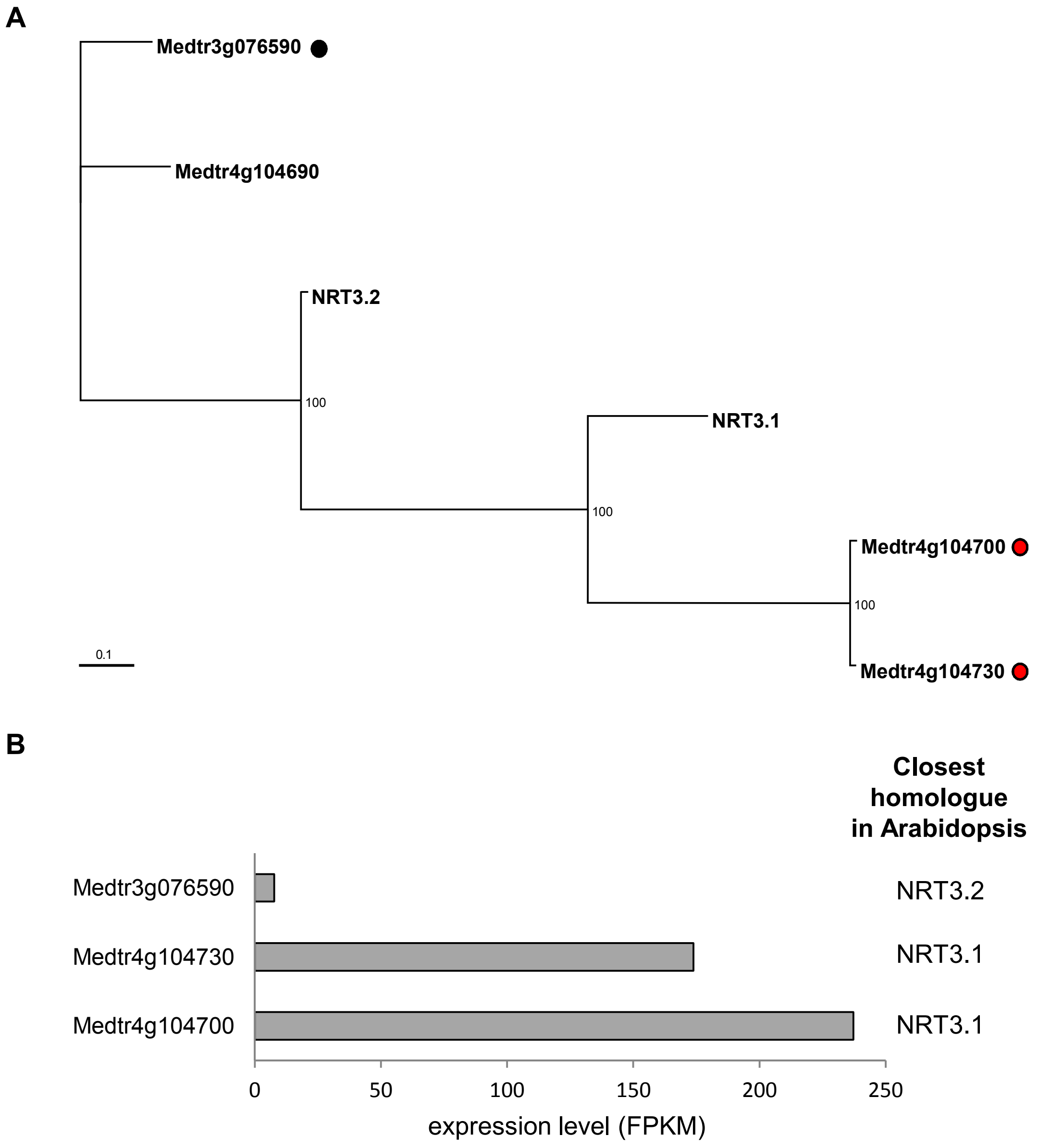


**Figure S7: The NRT3 nitrate transporter family in *Medicago truncatula* and its members displaying expression in root hairs.** (**A**) Phylogenetic relationship of *A. thaliana* and *M. truncatula* NRT3 proteins. Genes expressed in root hairs (FPKM value ≥ 1; see Table S2) are labeled with colored circles: red and black circles correspond to genes induced or not regulated by the NF treatments, respectively. (**B**) Relative expression (in FPKM) of *M. truncatula NRT3* genes in control root hairs.

A

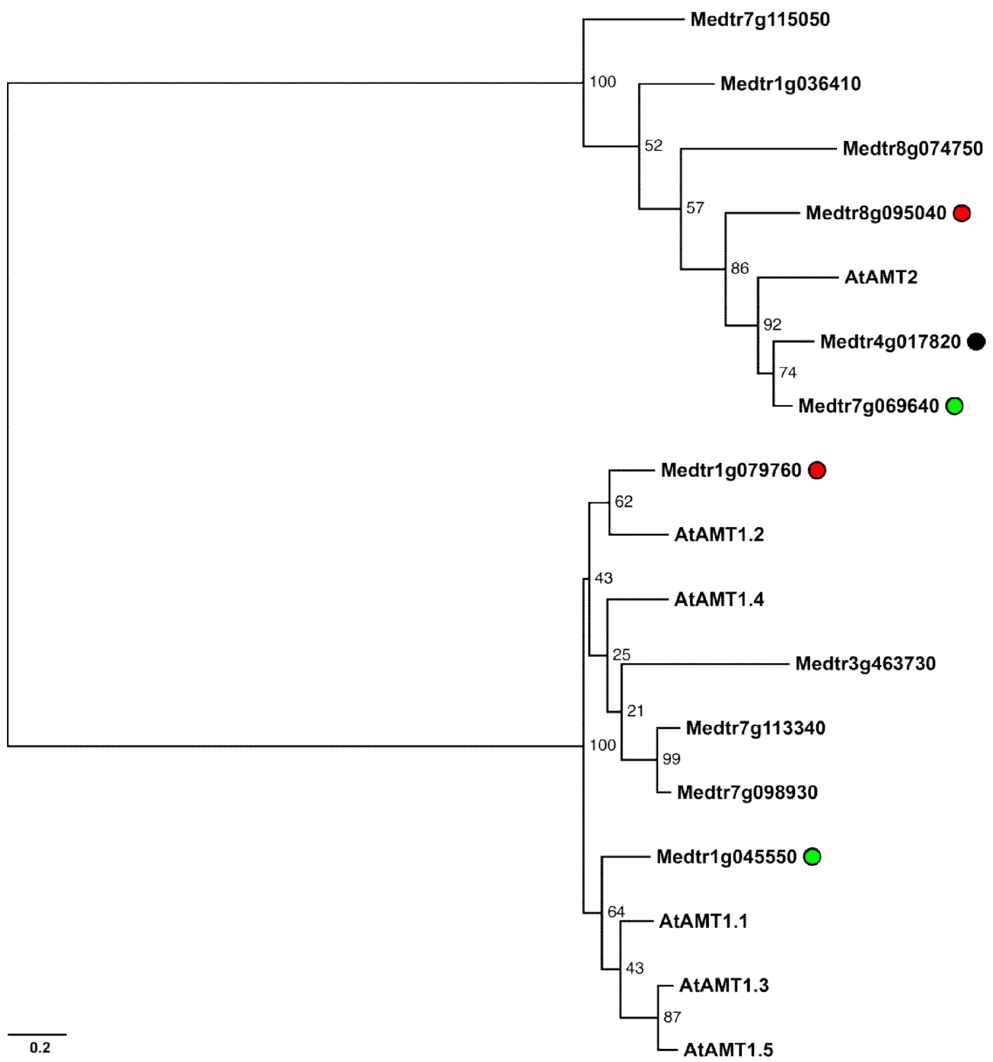


B

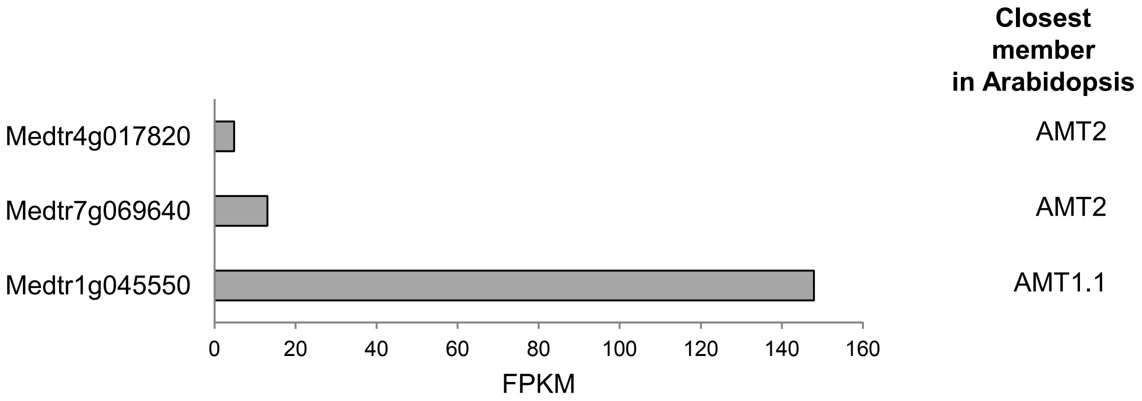


**Figure S8: The AMT ammonium transporter family in *Medicago truncatula* and its members displaying expression in root hairs.** (**A**) Phylogenetic relationship of *A. thaliana* and *M. truncatula* AMT proteins. Genes expressed in root hairs (FPKM value ≥ 1; see Table S2) are labeled with colored circles: red, green and black circles correspond to genes induced, repressed or not regulated by the NF treatments, respectively. (**B**) Relative expression (in FPKM) of *M. truncatula AMT* genes in control root hairs.

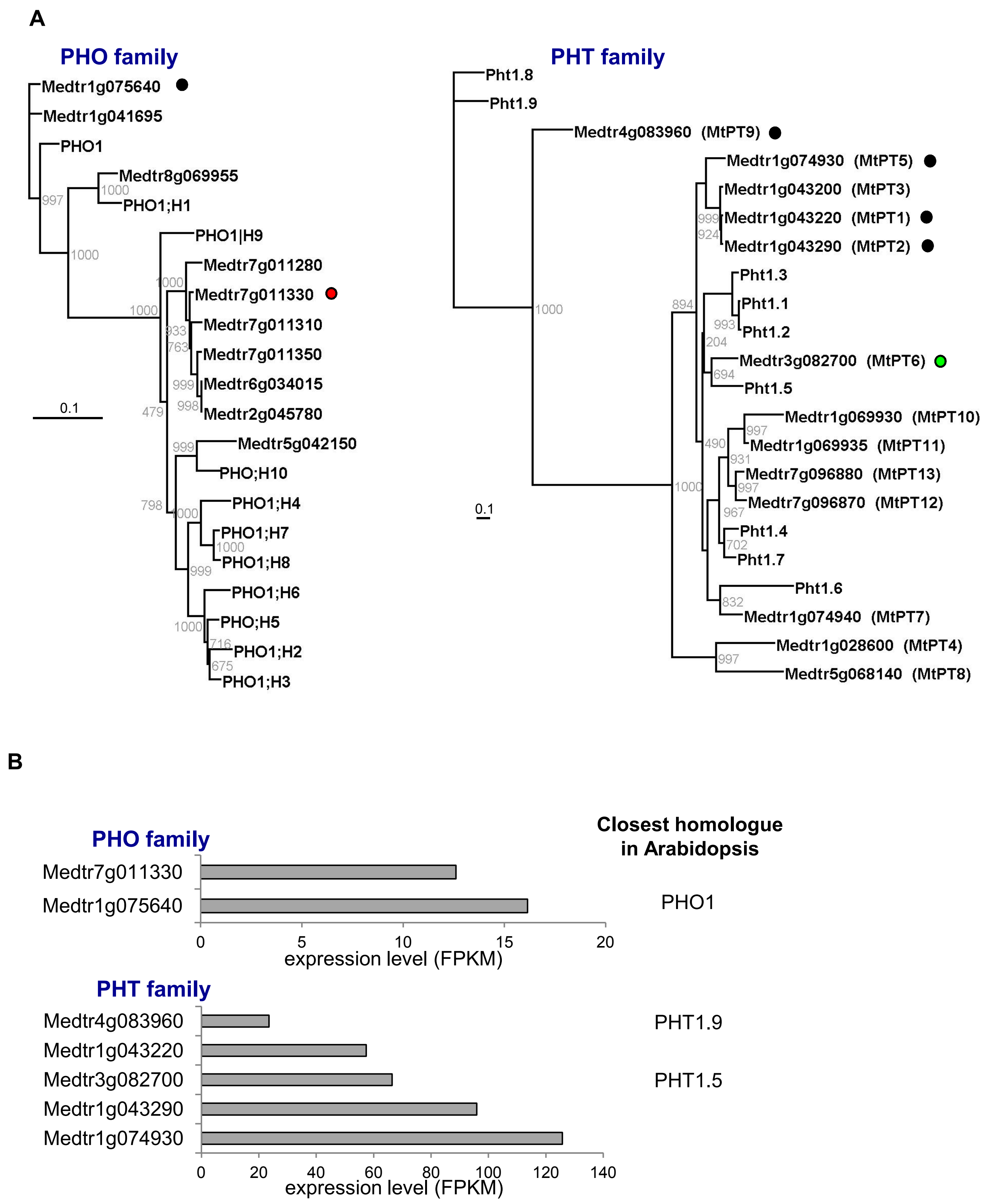


**Figure S9: The PHT1 and PHO1 phosphate transporter families in *Medicago truncatula* and their members displaying expression in root hairs.** (**A**) Phylogenetic relationship of *A. thaliana* and *M. truncatula* PHO1 (left panel) and PHT1 (right panel) proteins. Genes expressed in root hairs (FPKM value ≥ 1; see Table S2) are labeled with colored circles: red, green and black circles correspond to genes induced, repressed or not regulated by the NF treatments, respectively. (**B**) Relative expression (expressed in FPKM) of *M. truncatula PHO1* (upper panel) and *PHT1* (lower panel) genes in control root hairs.

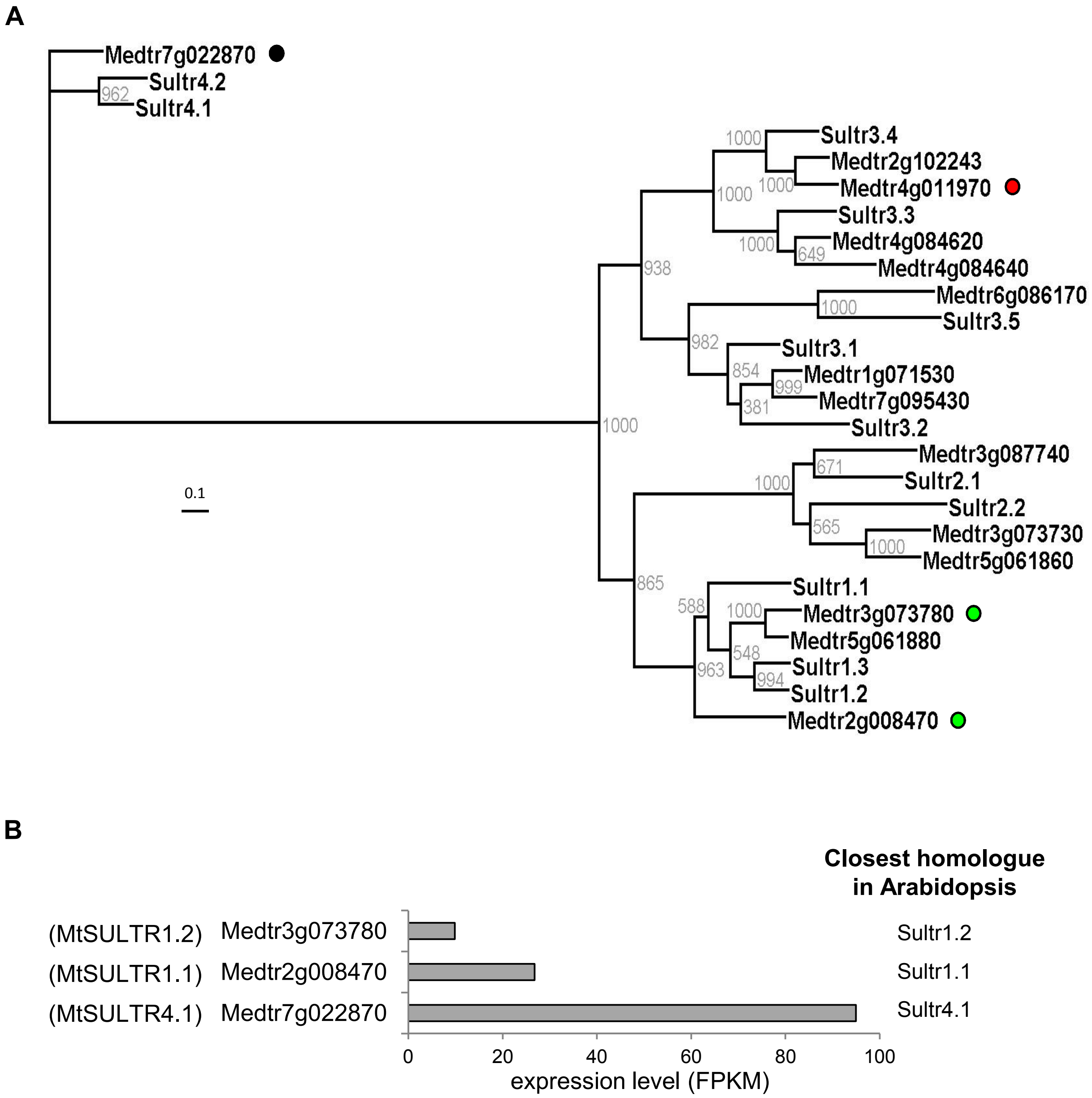


**Figure S10: The SULTR sulfate transporter family in *Medicago truncatula* and its members displaying expression in root hairs.** (**A**) Phylogenetic relationship of *A. thaliana* and *M. truncatula* SULTR proteins. Genes expressed in root hairs (FPKM value ≥ 1; see Table S2) are labeled with colored circles: red, green and black circles correspond to genes induced, repressed or not regulated by the NF treatments, respectively. (**B**) Relative expression (in FPKM) of *M. truncatula SULTR* genes in control root hairs.

A

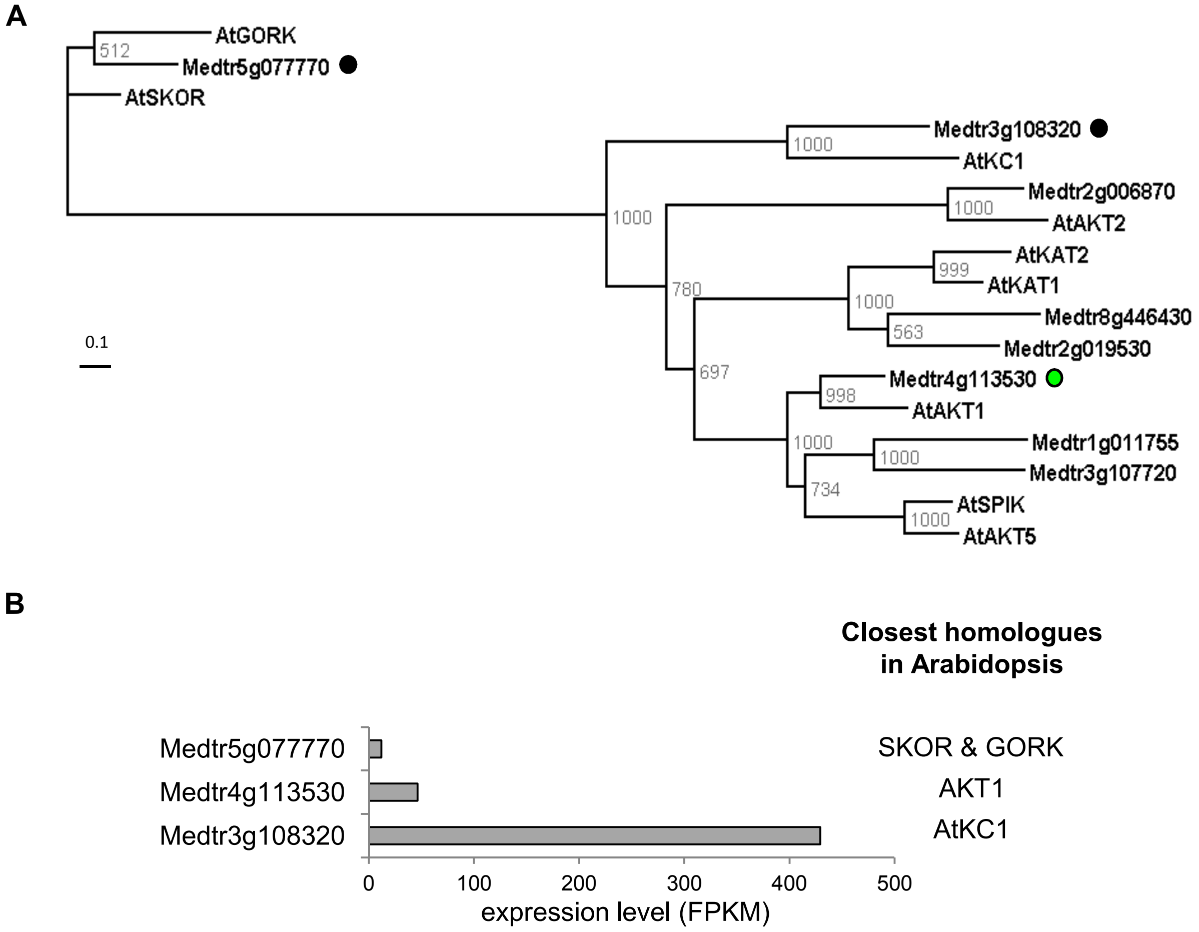


**Figure S11: The Shaker potassium channel family in *Medicago truncatula* and its members displaying expression in root hairs.** (**A**) Phylogenetic relationship of *A. thaliana* and *M. truncatula* Shaker proteins. Genes expressed in root hairs (FPKM value ≥ 1; see Table S2) are labeled with colored circles: green and black circles correspond to genes repressed or not regulated by the NF treatments, respectively. (**B**) Relative expression (in FPKM) of *M. truncatula Shaker* genes in control root hairs.

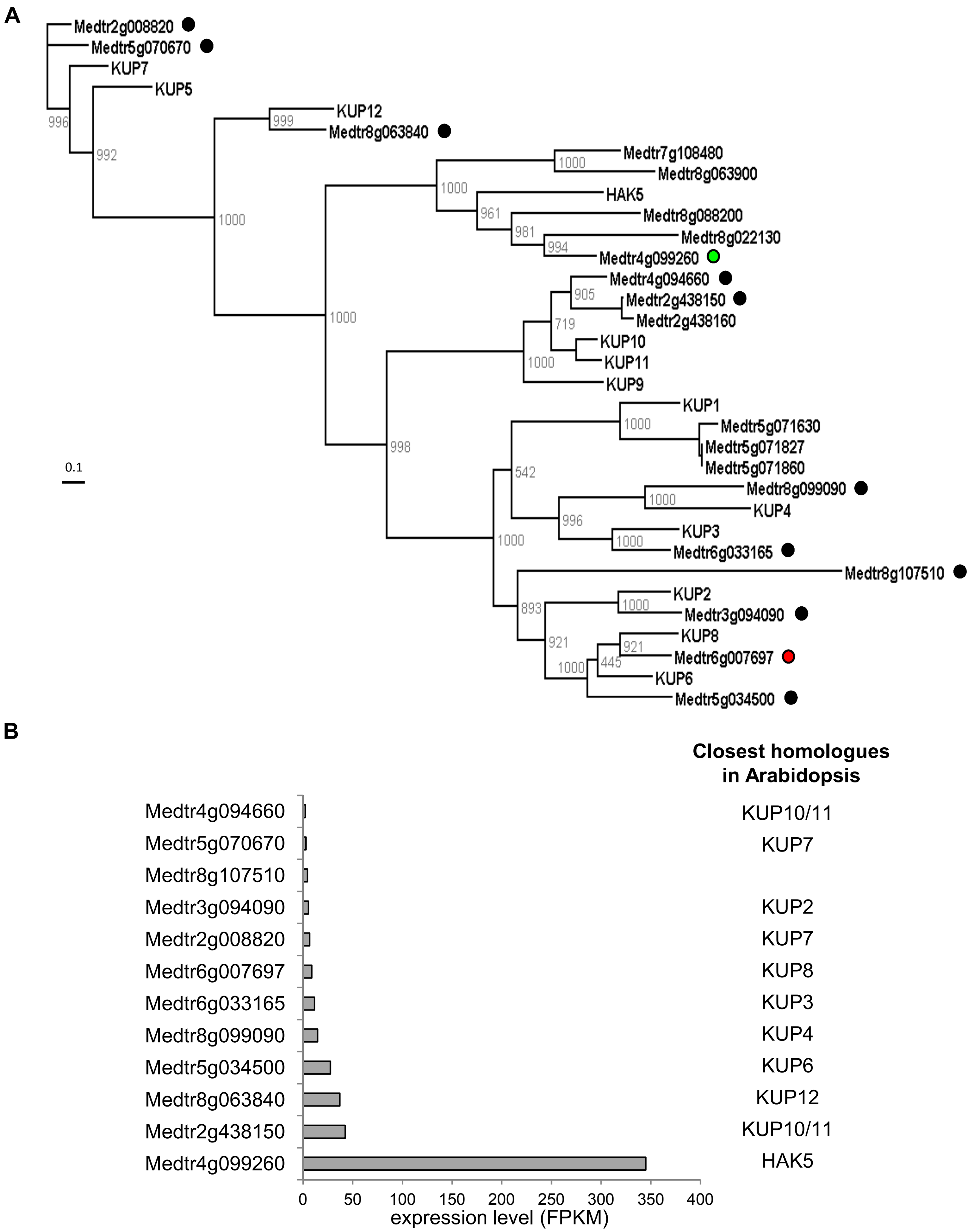


**Figure S12: The HAK/KUP/KT potassium transporter family in *Medicago truncatula* genome and its members displaying expression in root hairs.** (**A**) Phylogenetic relationship of *A. thaliana* and *M. truncatula* HAK/KUP/KT proteins. Genes expressed in root hairs (FPKM value ≥ 1; see Table S2) are labeled with colored circles: red, green and black circles correspond to genes induced, repressed or not regulated by the NF treatments, respectively. (**B**) Relative expression (in FPKM) of *M. truncatula HAK/KUP/KT* genes in control root hairs.

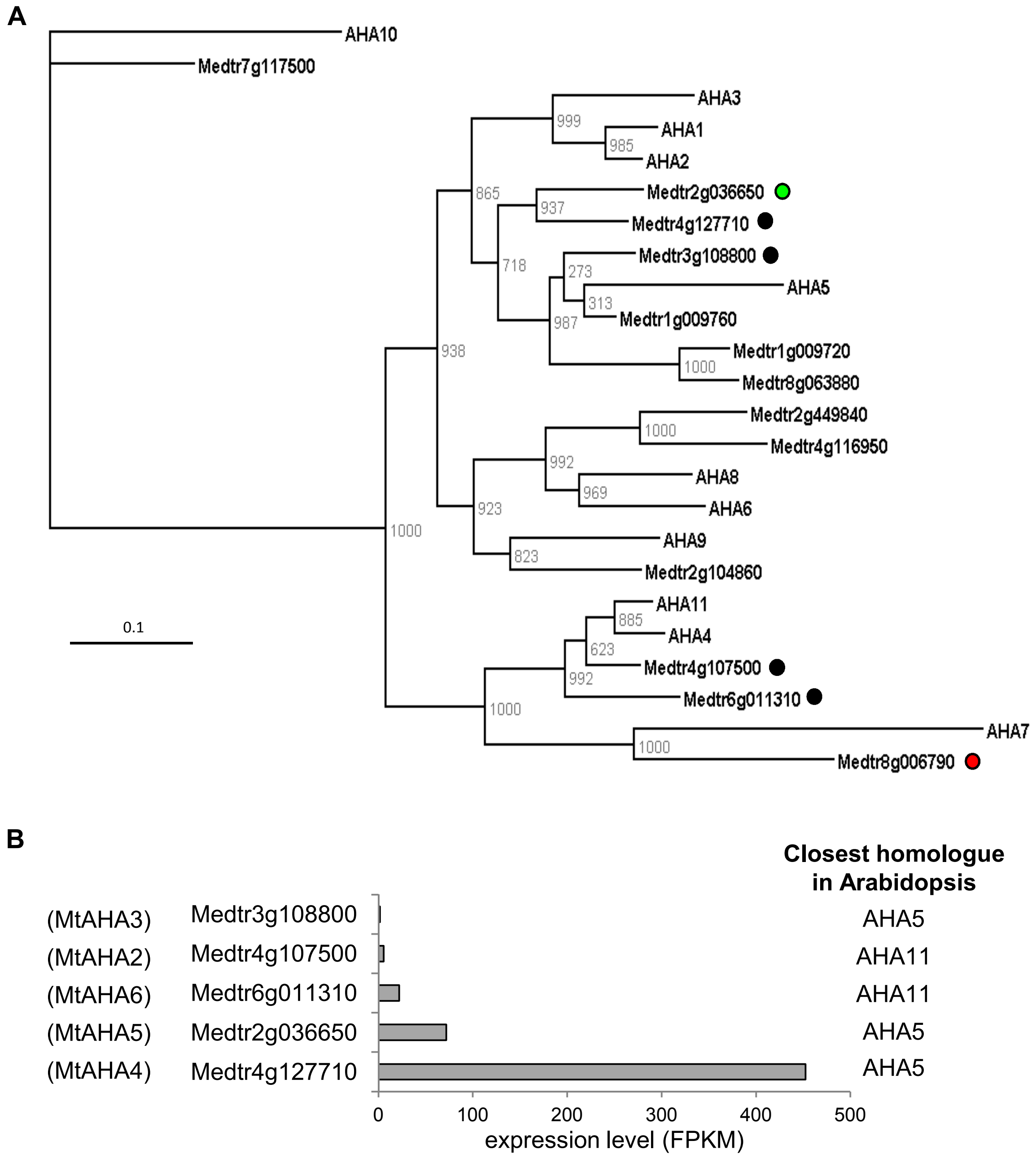


**Figure S13: The AHA proton pump family in *Medicago truncatula* and its members displaying expression in root hairs.** (**A**) Phylogenetic relationship of *A. thaliana* and *M. truncatula* AHA proteins. Genes expressed in root hairs (FPKM value ≥ 1; see Table S2) are labeled with colored circles: red, green and black circles correspond to genes induced, repressed or not regulated by the NF treatments, respectively. (**B**) Relative expression (in FPKM) of *M. truncatula AHA* genes in control root hairs.

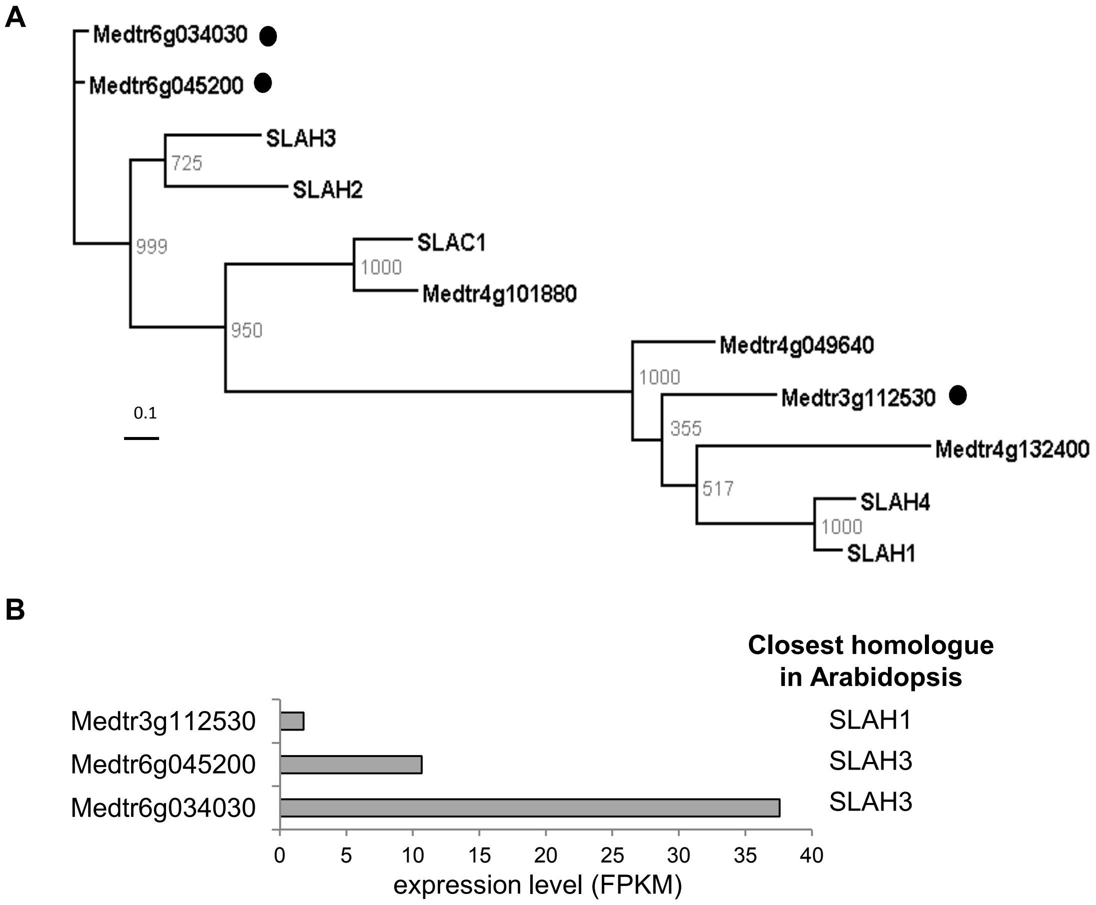


**Figure S14: The SLAC anion channel family in *Medicago truncatula* and its members displaying expression in root hairs.** (**A**) Phylogenetic relationship of *A. thaliana* and *M. truncatula* SLAC proteins. Genes expressed in root hairs (FPKM value ≥ 1; see Table S2) are labeled with black circles (not regulated by NF treatments). **(B)** Relative expression (in FPKM) of *M. truncatula SLAC* genes in control root hairs.

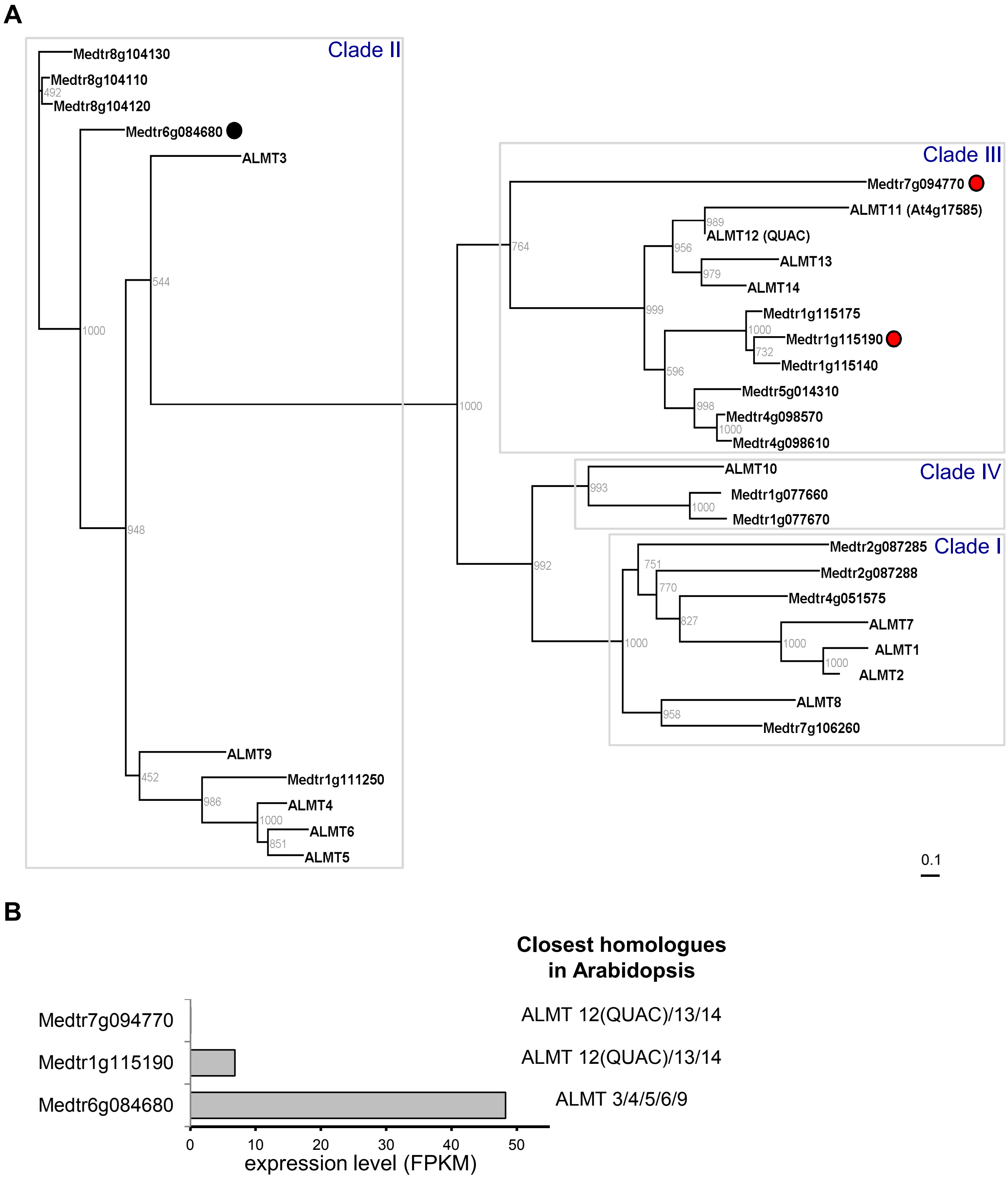


**Figure S15: The ALMT anion channel family in *Medicago truncatula* and its members displaying expression in root hairs.** (**A**) Phylogenetic relationship of *A. thaliana* and *M. truncatula* ALMT channels. Genes expressed in root hairs (FPKM value ≥ 1; see Table S2) are labeled with colored circles: red and black circles correspond to genes induced not regulated by the NF treatments, respectively. (**B**) Relative expression (in FPKM) of *M. truncatula ALMT* genes in control root hairs.

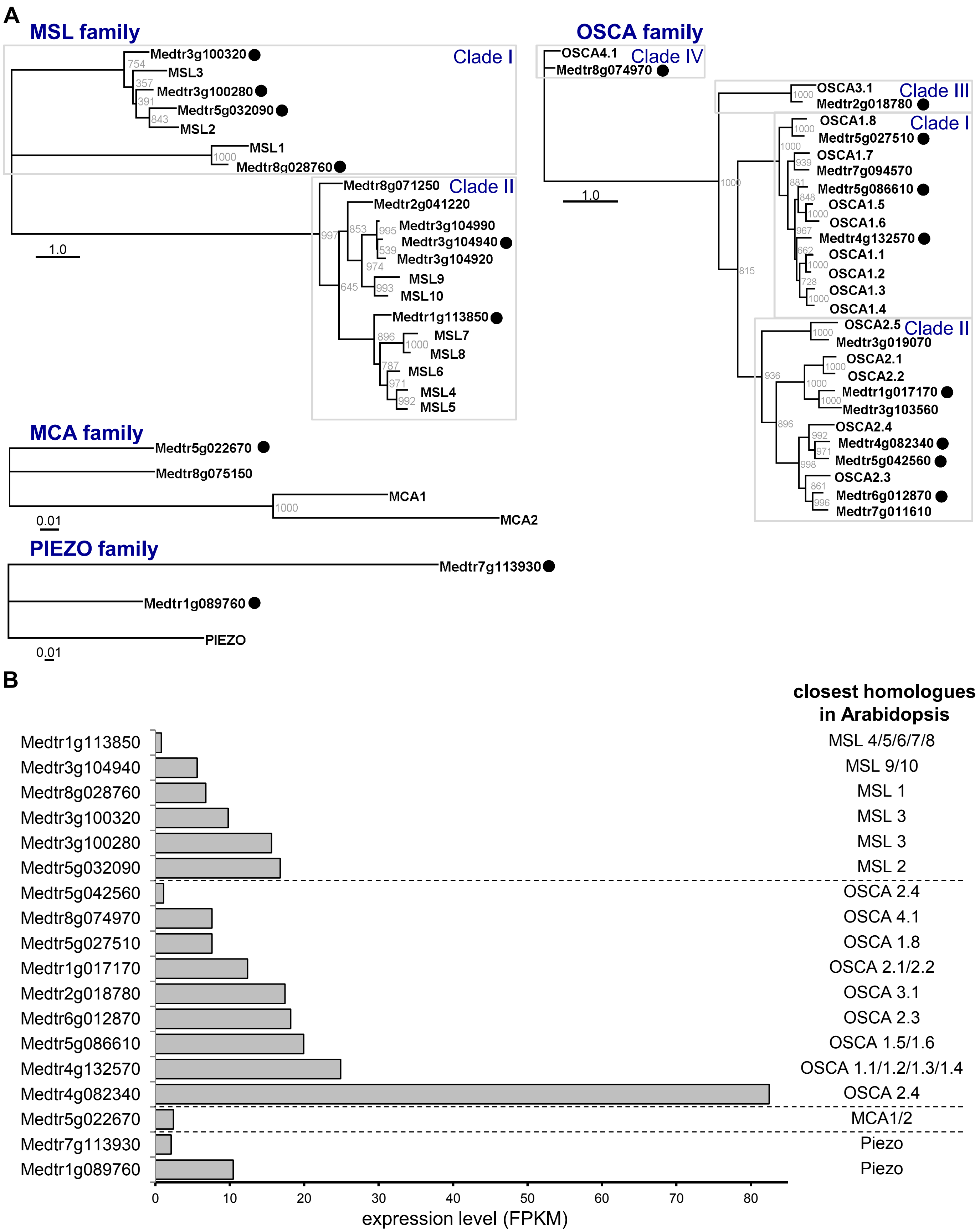


**Figure S16: Mechanosensitive and osmosensitive channels identified in the *Medicago truncatula* genome and its members displaying expression in root hairs.** (A) Phylogenetic relationship of MSL (upper left panel), OSCA (upper right panel), MCA (middle panel) and PIEZO (lower panel) channels in *A. thaliana* and *M. truncatula*. Genes expressed in root hairs (FPKM value ≥ 1; see Table S2) are labeled with black circles (not regulated by NF treatments). (**B**) Relative expression (in FPKM) of *M. truncatula MSL*, *OSCA*, *MCA* and *Piezo* genes in control root hairs.

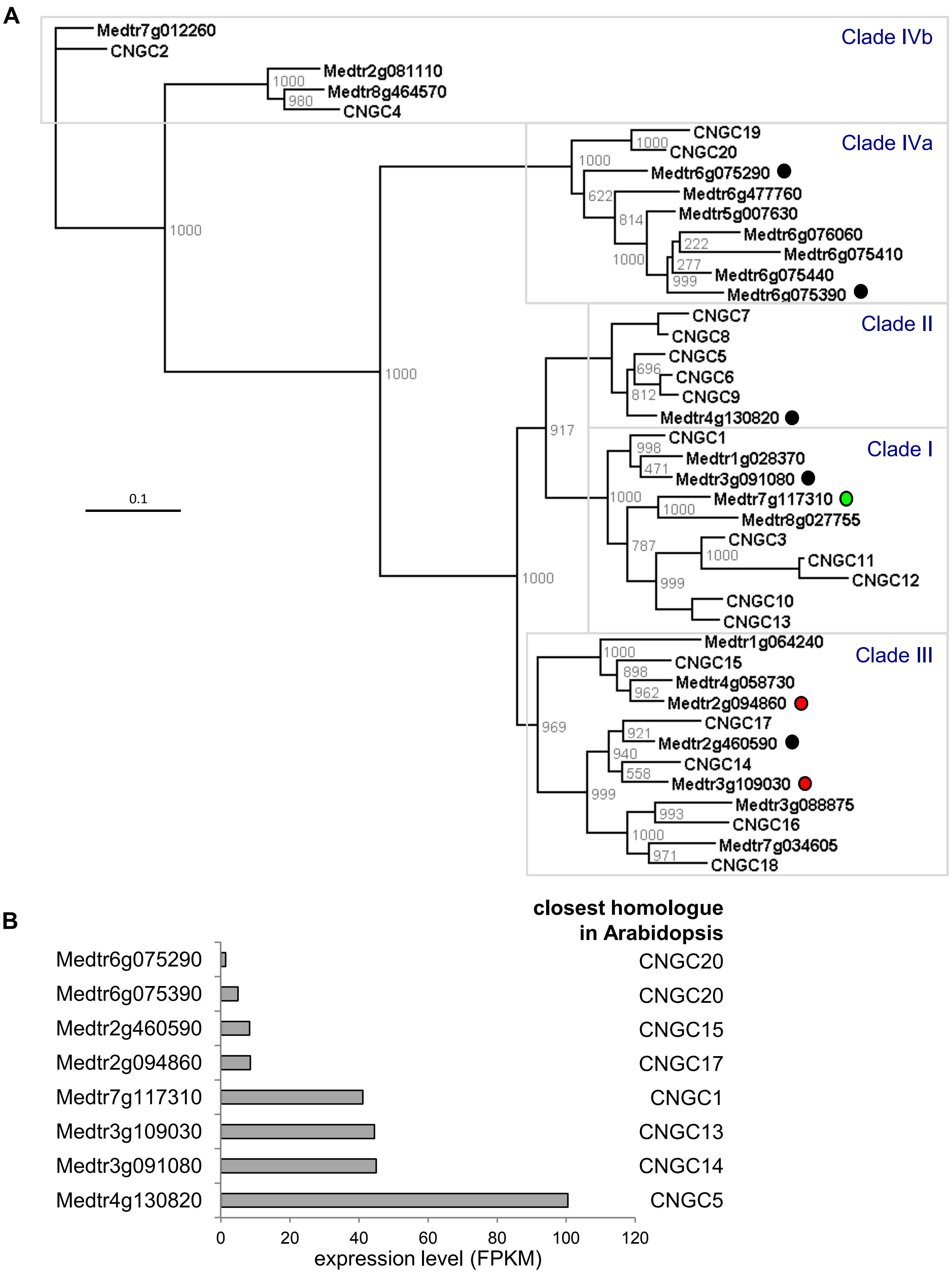


**Figure S17: The CNGC ion channel family in *Medicago truncatula* and its members displaying expression in root hairs.** (**A**) Phylogenetic relationship of *A. thaliana* and *M. truncatula* CNGC proteins. Genes expressed in root hairs (FPKM value ≥ 1; see Table S2) are labeled with colored circles: red, green and black circles correspond to genes induced, repressed or not regulated by the NF treatments, respectively. (**B**) Relative expression (in FPKM) of *M. truncatula CNGC* genes in control root hairs.

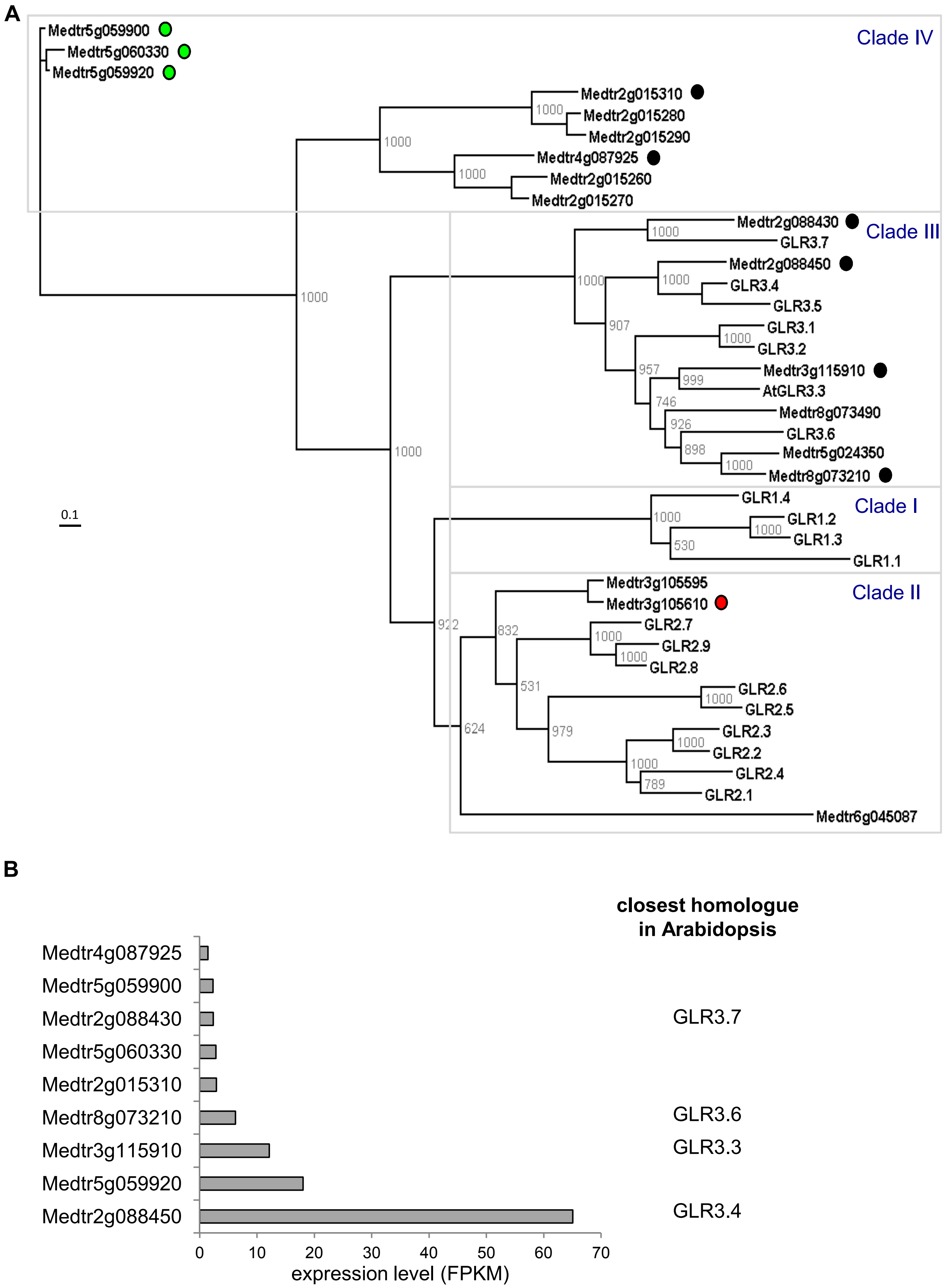


**Figure S18: The GLR ion channel family in *Medicago truncatula* and its members displaying expression in root hairs.** (**A**) Phylogenetic relationship of *A. thaliana* and *M. truncatula* GLR proteins. Genes expressed in root hairs (FPKM value ≥ 1; see Table S2) are labeled with colored circles: red, green and black circles correspond to genes induced, repressed or not regulated by the NF treatments, respectively. **(B)** Relative expression (in FPKM) of *M. truncatula GLR* genes in control root hairs.

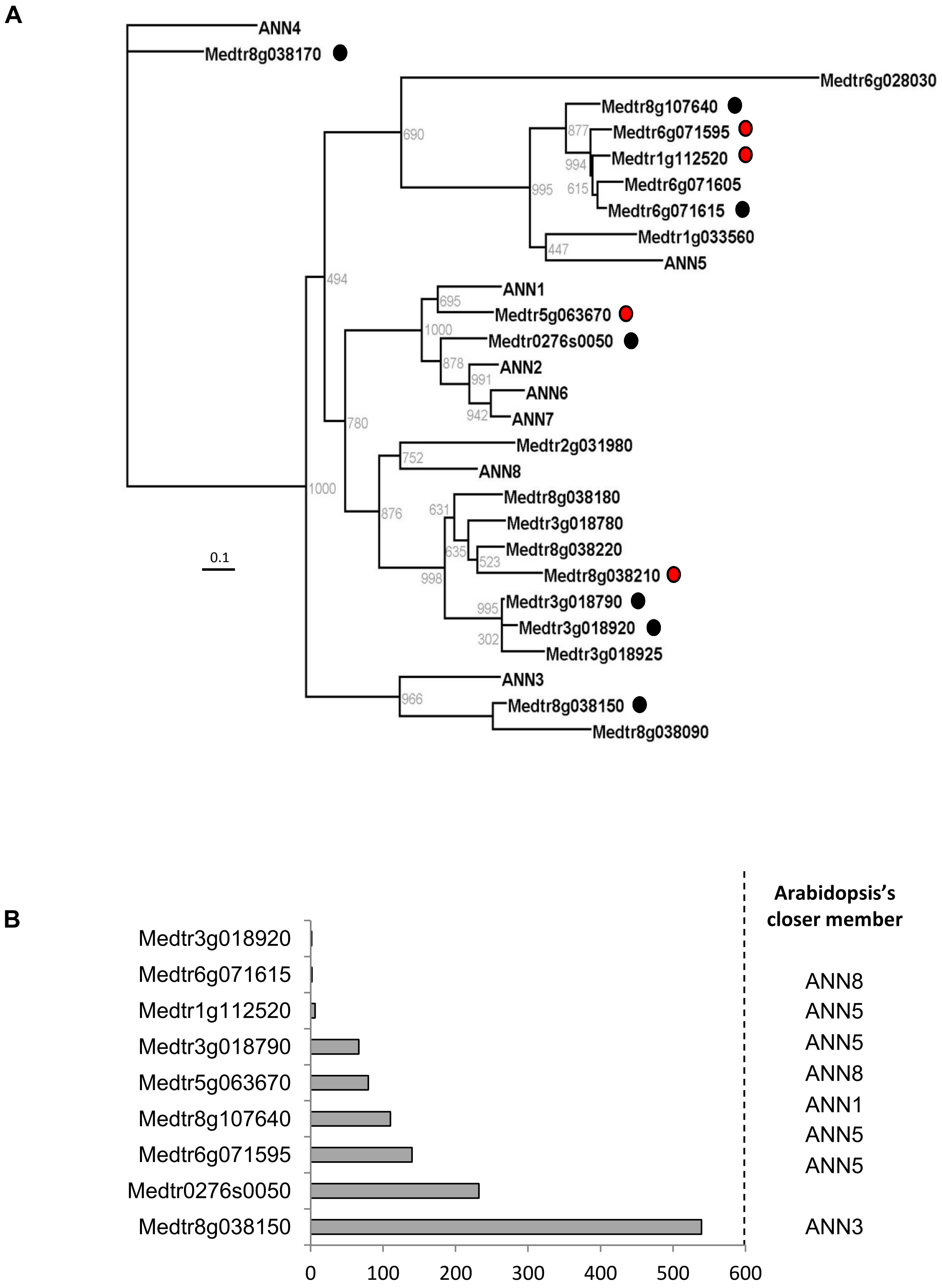


**Figure S19: The ANN gene family in *Medicago truncatula* and its members displaying expression in root hairs. (A)** Phylogenetic relationship of *A. thaliana* and *M. truncatula* ANN proteins. Genes expressed in root hairs (FPKM value ≥ 1; see Table S2) are labeled with colored circles: red and black circles correspond to genes induced or not regulated by the NF treatments, respectively. **(B)** Relative expression (in FPKM) of *M. truncatula ANN* genes in control root hairs.

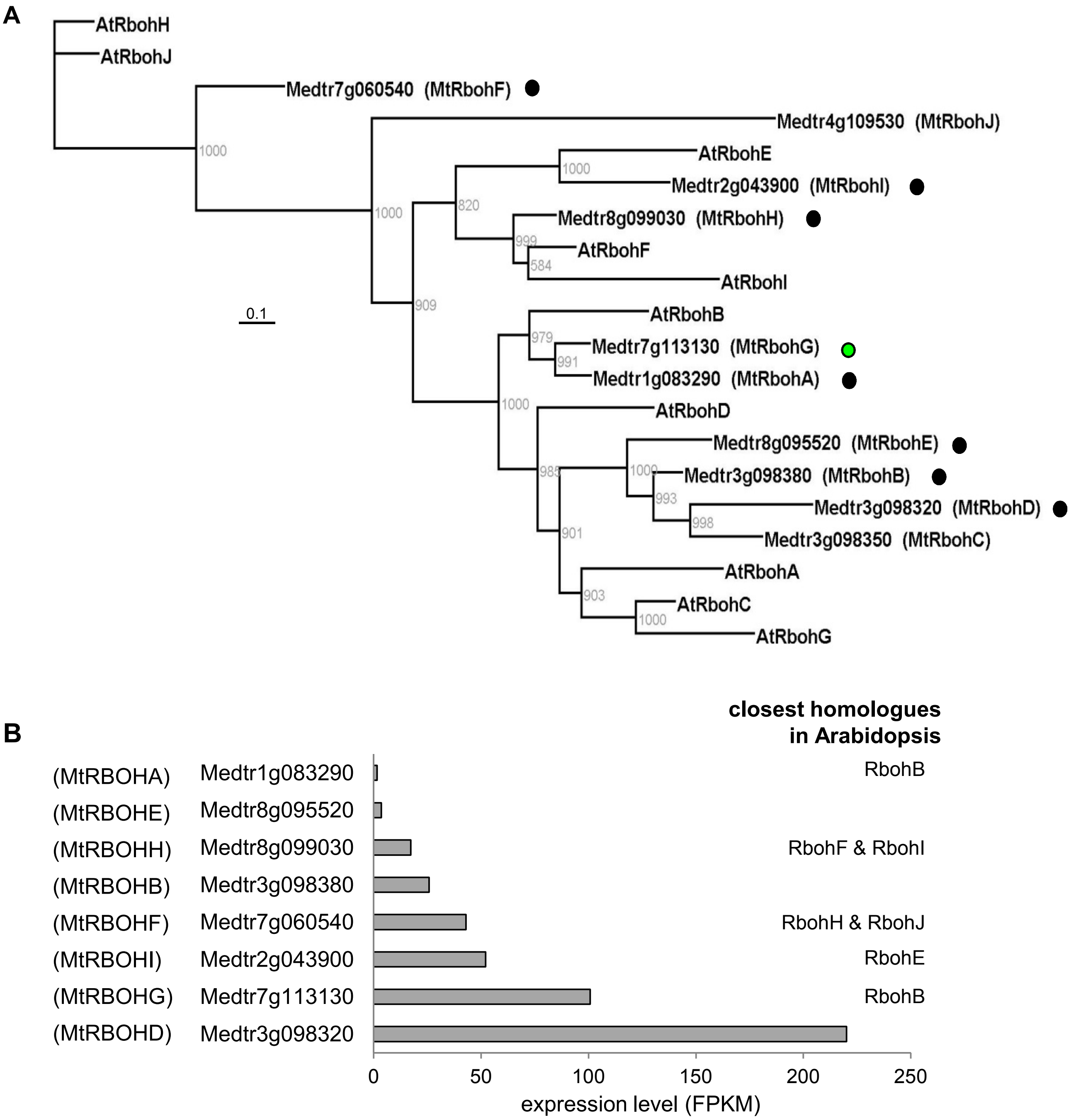


**Figure S20: The NADPH oxidase family in *Medicago truncatula* and its members displaying expression in root hairs.** (**A**) Phylogenetic relationship of *A. thaliana* and *M. truncatula* RBOH proteins. Genes expressed in root hairs (FPKM value ≥ 1; see Table S2) are labeled with colored circles: green and black circles correspond to genes repressed or not regulated by the NF treatments, respectively. (**B**) Relative expression (in FPKM) of *M. truncatula RBOH* genes in control root hairs.

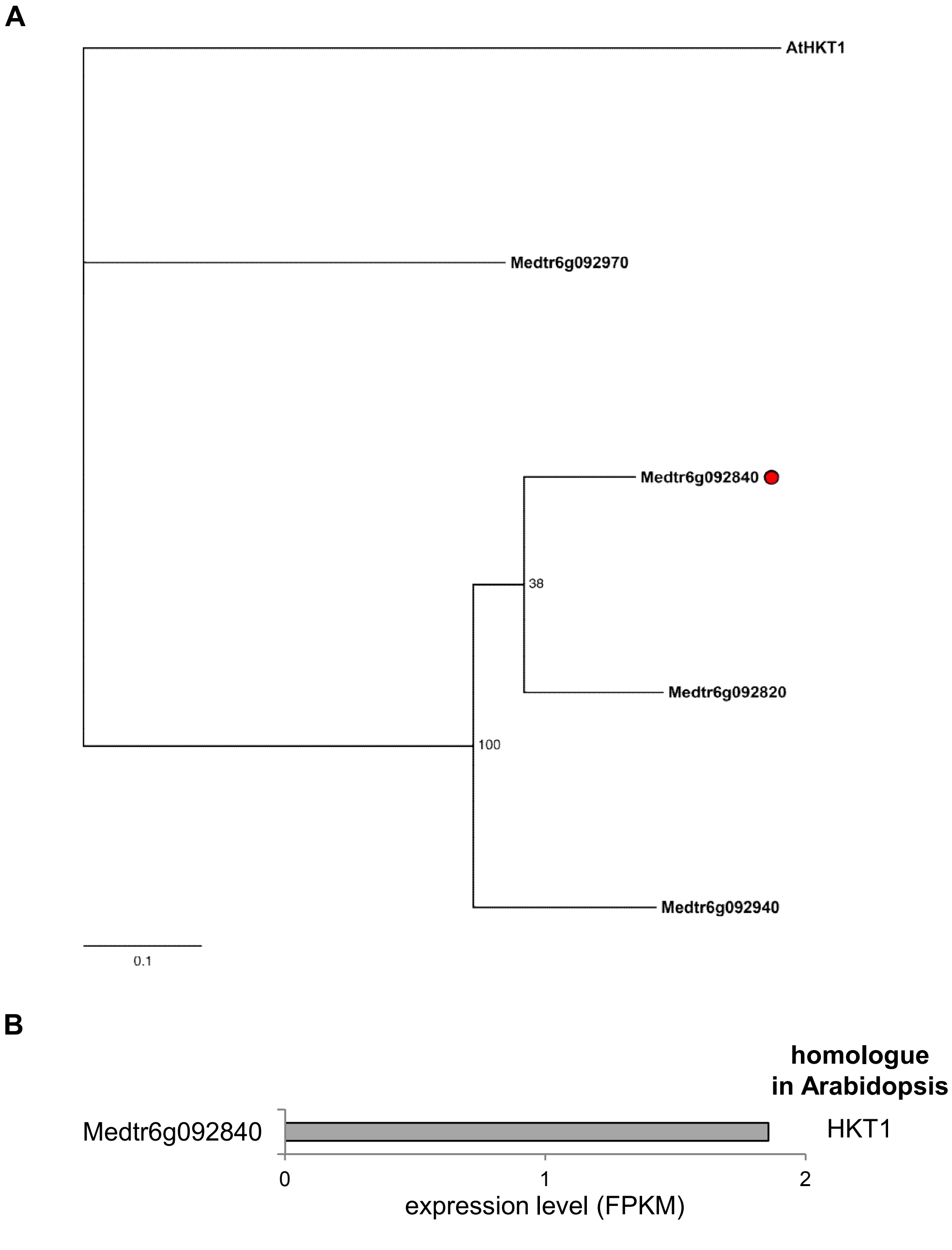


**Figure S21: The HKT transporter family in *Medicago truncatula*. (A)** Phylogenetic relationship of, *A. thaliana* and *M. truncatula* HKT proteins. Red circle: gene expressed in root hairs (>1 FPKM). This gene is induced by NF treatment. **(B)** Relative expression (in FPKM) of this gene in control root hairs.
